# Militancy in the military: military service and support for political violence and right-wing extremism

**DOI:** 10.1186/s40621-025-00626-5

**Published:** 2025-11-26

**Authors:** Elizabeth A. Tomsich, Garen J. Wintemute

**Affiliations:** Department of Emergency Medicine, Centers for Violence Prevention, University of California, 4301 X St, Sacramento, CA 95817 USA

**Keywords:** Military, Extremism, Political violence

## Abstract

**Background:**

Political violence constitutes an increasing threat to individual and population-level health in the United States, with military service identified as a potential risk factor. The current study examines the association between military service, combat experience, and support for and willingness to engage in political violence and approval of extremist organizations and movements.

**Methods:**

A nationally representative sample of 12,947 US adult members of the Ipsos KnowledgePanel completed the 2022 Life in America Survey. Outcomes are presented as weighted proportions and adjusted prevalence differences. The analytic sample comprised 2,255 respondents with military backgrounds; 1,105, including an augment of 415 respondents, reported combat experience.

**Results:**

Military respondents were less likely than others to perceive the use of political violence “to keep our borders open” as usually or always justified (adjusted prevalence difference (aPD) -3.1%, 95% confidence interval (CI) -5.6, -0.7, *q* = 0.049). By contrast, they demonstrated a greater willingness to “use force or violence on your own as an individual” (aPD 5.0%, 95% CI 2.5%, 7.5%, *q* = .001) and to “organize a group of people who share your beliefs to use force or violence” (aPD 2.8%, 95% CI 0.7%, 4.8%, *q* = .029) to advance a political objective. They were also more likely to report that it would be very or extremely likely that they would be armed with a gun (aPD 6.3%, 95% CI 3.5%, 9.2%, *q* = .001) or carry a gun openly (aPD 6.5%, 95% CI 3.1%, 10.0%, *q* = .001) in a situation where they thought force or violence was justified to advance an important political objective; however, there were no differences with respect to threatening or shooting someone with a gun. Additionally, military respondents were more likely to strongly or very strongly approve of the Oath Keepers (aPD 4.1%, 95% CI 0.7%, 7.6%, *q* = .037). There were no significant differences by combat experience.

**Conclusions:**

Given the modest number of differences among numerous comparisons, and the relatively small size of prevalence differences, it does not appear that military service and combat experience act as risk factors for support for and willingness to engage in political violence, or approval of extremist organizations and movements.

**Supplementary Information:**

The online version contains supplementary material available at 10.1186/s40621-025-00626-5.

## Introduction

The January 6th, 2021 insurrection exposed the stark reality of political violence in the United States. Concurrently, disquieting findings from public opinion polling [14, 46, 48], survey research [30, 58], and the report of the House Select Committee to Investigate the January 6th Attack on the United States Capitol [24] indicate democracy may be increasingly under duress. Evidence-based intervention against this threat to individual and population-level health [63] requires an understanding of which populations may be at greater risk for political violence, as well as the circumstances that elicit the use of force or violence for political objectives. A widely circulated report examining those charged for their role in the Capitol Hill siege focused on one factor disproportionately present among arrestees relative to the general population—military experience [38]. Whereas 7% of American adults are military veterans [57] and 0.4% are on active duty [44], approximately 15% of defendants charged for their involvement in the insurrection have a military background [40]. The aim of this study is to investigate links between military service and political violence.

## Background

The prevalence of veterans and service members among January 6th defendants may reflect a long history of problems with extremism in the military community [38]. Combat veterans played a role in the rise of the white power and militia movements after the Vietnam War [7, 38], and an examination of extremism in the military over the past two decades documented issues such as physical harm to other service members and/or civilians due to hate crimes or violence by extremists,theft of military equipment for purposes of arming or funding extremists,the illegal provision of intelligence and information related to explosives and weapons of mass destruction to other extremists,and harms to mission success, morale, unit cohesion, and the recruitment and retention of minoritized service members [3].

More recently, the rise of the alt-right and the emergence of groups such as the Oath Keepers, a right-wing anti-government association of active and former law enforcement, first responders, and military service members, have contributed to increases in extremist activities in the military [3]. In addition to recruiting individuals with a military background, domestic extremist/terror groups have been documented as encouraging members to enlist in the military in order to acquire combat experience and tactical training [16, 47].

### Scope of the problem

Governments in the United States have long recognized the hazard posed by private military organizations. All 50 states have laws restricting paramilitary activity, or militias characterized by shared political beliefs that operate outside of the government under a hierarchical command structure, use military-type uniforms and tactical gear, and engage in armed drills [35]. Experts suggest that veterans and active-duty military personnel may comprise approximately 25% of active militia members [53].

Research also indicates individuals with military backgrounds may be disproportionately represented among individuals who commit crimes motivated by extremist views or affiliations relative to the general population. In a sample of offenders who committed such crimes in the United States between 1990 and August 2023, 15.1% had a military background [28].

This problem has increased over time. Between 1990 and 2010, an average of 7.1 individuals who had military backgrounds committed criminal offenses based on extremist views or affiliations per year; since 2011, the average has increased by over five times to 39.1 individuals per year [28]. Representation has been particularly high since 2017, with an average of 63.3 per year [28]. An even higher proportion are involved in the most extreme of attacks. Around 1 in 4 individuals involved in mass casualty terrorist plots or attacks between 1990 and 2022 had military backgrounds [27]. A multivariate analysis revealed that among all individuals involved in extremist crimes in the U.S. between 1990 and 2022, having a U.S. military background was the strongest predictor of involvement in a mass casualty plot or attack [27].

Seventy four percent of mass casualty plotters or offenders with a military background were associated with far-right domestic extremist groups and movements; just under 40% held white supremacist/nativist views and/or were affiliated with neo-Nazi groups [27]. Reports from military personnel suggest that far-right extremism in the general population of the armed forces may be increasing–a 2019 survey of active-duty troops found that 36% reported witnessing white nationalist or racist views or behavior within the military—up from 22% in 2018, rates were even higher (53%) among respondents of color [50].

Despite these links between military background and extremism, there appears to be only two studies of the endorsement of political violence among a general population of service members and veterans. The RAND Corporation conducted a nationally representative survey of almost 1,000 veterans in the U.S. [22]. Results indicated generally lower rates of support for extremist ideologies and groups among veterans compared to rates generated from prior representative surveys of the general population, including white supremacism (0.8% vs 7.0%) and the Proud Boys (4.2% vs 9.0%). Relative to the general population, support for QAnon (a far-right conspiracy theory alleging the world is controlled by a secret cabal of Democratic Satan-worshipping pedophiles) (13.5% vs 17.0%) and the Great Replacement theory (the belief that left-leaning elites are intentionally replacing native-born populations with immigrants for political gain) (28.8% vs 34.0%) among veterans were lower, while support for political violence (17.7% vs 19.0%) was similar. Conversely, Pape et al. [43] found that veterans were nearly twice as likely as non-veterans to endorse high insurrectionist statements (“The use of force is justified to restore Donald Trump to the presidency” , “I would personally use force to restore Donald Trump to the presidency”) in a nationally representative survey conducted in 2021 and 2022.

Combat experience and its psychological sequelae have been identified as potential sources of susceptibility for recruitment into extremist groups or movements [29, 34]. The rate of deployment to a combat zone or a location in support of combat operations identified among January 6th arrestees (44%) [38] was somewhat higher than that reported by a probability-based sample of veterans in a 2019 survey (36%) [45]. However, in a larger sample of individuals with military backgrounds who committed criminal acts motivated by political, economic, social, or religious goals between 1990 and 2021, only 30% had been deployed to an active combat zone [29]. Among extremist offenders with a military background, the rate of combat experience (19%) was lower than that observed in a general population of veterans (29%) [29, 45]. Experts have called for further investigation into the relationship between extremism, anti-government sentiment, and combat experience [38].

### Proposed mechanisms of radicalization among service members and veterans

Consistent with theories of informal social control, research on military service identifies it in some respects as protective, providing economic security, occupational status, and job stability, as well as supporting desistence from crime [9, 10, 49]. However, research identifying military experience as a potential risk factor for terrorism [52] suggests that military service may not function as a social bond that constrains political violence [11].

Research on extremism among military service members and veterans proposes several push and pull factors. Some center on the culture of the military and the psychological processes associated with the transformation into a soldier, such as active recruitment, indoctrination, group solidarity and polarization, isolation from opposing viewpoints, a sense of vicarious justice, excitement, glory, hypermasculinity, and social learning of and desensitization towards violence, with experts noting the similarities between the mechanisms of martialization and radicalization [1, 4, 19, 21, 39]. Others have suggested that enlistment screening is inadequate, particularly since 9/11, when recruitment and retention standards weakened to meet intensified enlistment goals and address concerns with respect to attrition, facilitating the infiltration of white supremacists and other extremists into the military [12, 17, 20, 23, 31].

Given that most extremists with a military background are veterans, the transition toward civilian life may function as a pull factor [52], due to “unfreezing” [36, 39], or the loss of community, purpose, and belonging [11], factors that typically constrain or “freeze” people to conform with the rules, expectations, and values of significant people and institutions in their lives. Fundamental uncertainty with respect to oneself or the future and a loss of meaning serve as vulnerabilities to extremist radicalization [18]. The loss of significance—a predictor of ideologically motivated violence [26]–constitutes another potential consequence of exiting the military, triggered by identity discrepancies resulting from involuntary role exits or the perception that achievements during enlistment are not recognized or appreciated [51]. As service members and veterans tend to be more conservative than those who have not served [42], and extremists with a military service background are more likely than those without to have affiliations with right-wing terrorist groups [5], the appeals of right-wing extremist groups and movements may be particularly attractive to those seeking to assuage these losses [11].

Other research comparing extremists with and without military experience found the latter more likely to experience trauma, mental illness, a diminution in social standing prior to radicalization, social, cultural, religious, or political ostracism and marginalization, challenges with initiating or maintaining romantic relationships, anger with the US, and a group-based grievance [21]. Military service, particularly combat experience, is characterized by extreme “physical and psychological risks and strains” (p. 526), which can increase risk for trauma and mental health issues and complicate relationships [21]. Likewise, involvement in a contested conflict or military campaign may engender feelings of ostracism and anger against the US. In the case of some Vietnam veterans, alienation from their country and a sense that the values they fought for had been betrayed pushed them into anti-government extremist groups [7].

### Current study

The current study contributes to the literature on military service and extremism by examining variation in beliefs about democracy and American society, support for political violence in different circumstances, personal willingness to engage in political violence, and approval for extremist right-wing organizations and movements in association with two exposures: military background and, among veterans and service members, combat experience.

## Methods

The 2022 Life in America Survey was designed by the authors and administered online by the survey research firm Ipsos in English and Spanish from May 13 to June 2, 2022 [60]. Forty KnowledgePanel members participated in a pretest of the English language version of the survey that was administered April 27 to May 2, 2022. Prior to starting the survey, respondents received the following informed consent language: “[by] continuing, you are agreeing to participate in this study.” The UC Davis Institutional Review Board approved the study, which is reported following American Association for Public Opinion Research guidelines [2].

### Participants

Respondents were recruited from the Ipsos KnowledgePanel, an online research panel widely used in population-based research, including studies of violence and firearm ownership [33, 37, 59]. An augment was used to increase the sample size of respondents with combat experience.

Ipsos establishes a nationally representative panel by recruiting members on an ongoing basis through address-based probability sampling using data from the US Postal Service’s Delivery Sequence File [25]. Panel members in households without internet access are provided a web-enabled device and free internet service. Ipsos uses a primarily points-based incentive program to encourage participation and promote participant retention in KnowledgePanel.

A study-specific sample was selected using a probability-proportional-to-size procedure. Panel members aged 18 years and older were eligible for selection. Invitations were sent by e-mail; after 3 days, non-respondents received automatic reminders by e-mail and telephone. Recruitment for respondents with combat experience remained open until a pre-specified sample size was achieved.

Ipsos provided a final survey weight variable that adjusted for the initial probability of selection into the survey, survey-specific nonresponse, and over- or under-coverage using design weights with post-stratification raking ratio adjustments. With weighting, the sample is designed to be statistically representative of the noninstitutionalized adult population of the US consistent with the 2021 March supplement of the Current Population Survey [25].

### Measures

Sociodemographic data, including information on military and combat service backgrounds, were collected by Ipsos from profiles created and maintained for each survey respondent. Military background was measured by an affirmative answer to one or more of the following questions: “Did you ever serve on active duty in the U.S. Armed Forces?,” “Are you now in the U.S. Armed Forces?,” “Have you ever been a member of the Reserve or National Guard?,” and “Are you currently a member of the Reserve or National Guard?” Combat experience was measured by the following question: “Did you ever serve in a combat or war zone? Persons serving in a combat or war zone often receive combat zone tax exclusion, Imminent Danger Pay, or Hostile Fire Pay.”

Survey questions analyzed in this study addressed 4 general domains: beliefs regarding democracy and the potential for violence in the US; beliefs about race and ethnicity and American society; endorsement of violence, including political violence; and support for extremist groups and movements, including the Proud Boys, Oath Keepers, Three Percenters, the militia movement, the white supremacy movement, the Christian nationalist movement, and the Boogaloo movement. Some questions were selected or adapted from prior surveys on these topics to enable monitoring of trends and provide context for findings on questions not previously asked.

To determine whether differences observed in support for political violence were specific to that subtype of violence, initial items on violence inquired about its use in non-political situations. These 7 questions were presented in a fixed order that, in the judgement of the authors, proceeded from more to less likely to be perceived by respondents as justifiable: from “in self-defense” to “to get respect”. This expected response transition from support to nonsupport for violence would subsequently need to be reversed for respondents to report support for political violence on successive questions.

Respondents were then asked about the degree to which they considered political violence to be justified “in general” and to advance 17 specified political objectives, such as “to return Donald Trump to the presidency this year,” “to stop people who do not share my beliefs from voting,” and “to stop a protest or demonstration.” Of the 17, nine were presented to all respondents. Eight additional objectives were paired, with each respondent receiving only 1 item from each pair, resulting in each respondent being presented with questions on 13 of 17 objectives.

Respondents who considered political violence to be at least sometimes justified were asked about their personal willingness to engage in political violence, by type of violence (to “damage property,” “threaten or intimidate a person,” “injure a person,” “kill a person”) and by target population (e.g. “an elected federal or state government official;” “a public health official;” “a person who does not share your race or ethnicity”). All respondents were asked about the likelihood of their future use of firearms in situations where political violence was justified (e.g., “I will be armed with a gun”; “I will shoot someone with a gun”). Questions on violence defined “force or violence” as “physical force strong enough that it could cause pain or injury to a person.” Questions on respondents’ support for and willingness to engage in political violence used the phrase “force or violence to advance an important political objective that you support”.

### Implementation

During implementation, respondents were randomized 1:1 to view response options from negative to positive valence (e.g., from ‘do not agree’ to ‘strongly agree’) or the reverse throughout the questionnaire. In questions with multiple statements, the order of the statements was randomized except in cases where ordering was necessary. Responses that might invoke a skip pattern included non-response prompts. The survey integrated several design elements intended to minimize inattentive responses and bias in reported support for political violence [61].

Response options were unipolar scales without a neutral midpoint (e.g., do not agree, somewhat agree, strongly agree, very strongly agree). While the use of neutral midpoints remains debated, we selected a unipolar format to minimize satisficing behavior [13]. In order to further minimize the potential for bias due to satisficing, the analysis emphasizes responses above the “somewhat” or “sometimes” level.

### Analysis

To generate prevalence estimates, we calculated weighted percentages and 95% confidence intervals (CI) using svy: tabulate in Stata version 15.0 (College Station, TX: StataCorp LLC). Outcomes were defined dichotomously to produce prevalence differences. Unadjusted and adjusted prevalence differences and their 95% confidence intervals (CI) were determined by using the margins command after running glm models in the Poisson family with a log link, with robust standard errors to correct for design effects and heteroskedasticity in binary outcomes. Two multivariable models were used to adjust for covariates: Model 1 included age, gender, and race and ethnicity; Model 2 retained those variables and added income, education, region, political party, and an additional conditionally relevant variable [15] that served as a proxy for measuring whether respondents with a military background may have been conscripted into service. Specifically, male respondents with military backgrounds who were born in 1952 or earlier were categorized as potential draftees [6]. We selected Model 2 as the final model based on theory, prior research, and fit statistics.

P-values were corrected for multiple comparisons by controlling the false discovery rate (FDR) using the Benjamini and Hochberg method [8]. The terminology for the corrected values is FDR-adjusted p-values or q-values [54], we use the latter term here. Q-values represent the probability that the given difference would be a false discovery, or the expected proportion of “false positives” that would be seen among the collection of all differences whose q-values were at or below the given q-value. The article text, tables, and figures present prevalence differences,prevalences are additionally in the figures and tables.

The primary analyses focus on military background as the exposure; in supplemental analyses we replicate the models among military respondents with combat experience as the exposure and examine associations between sociodemographic factors and strong or very strong agreement with the potential need for violence in the United States within 1) the military sample, and 2) the combat sample.

## Results

Of 22,853 panel members invited to participate, 12,947 qualified respondents completed the survey, yielding a 56.7% completion rate. Demographic descriptions of respondents by military background and combat experience are in Appendix Tables S1 and S2; descriptions of respondents and nonrespondents have been reported in other work [61]. Of the 12,947 respondents, an unweighted count of 2,255 identified military backgrounds (9.5%, 95% confidence interval (CI) 9.0%, 10.1%). An unweighted count of 1,105 respondents, including an augment of 415 respondents, reported combat experience (3.1%, 95% CI 2.8%, 3.3%). Among military respondents, most were veterans,5.8% (95% CI 4.1%, 8.1%) were active duty (n = 62).

### Democracy, the potential need for violence, and civil war

There were no significant differences between respondents with military backgrounds and non-military respondents on items measuring perspectives on democracy, the potential need for violence, and the prospect of civil war in the United States (Tables S3, S4).

### Race and ethnicity and american society

Military respondents were more likely than non-military respondents to strongly or very strongly agree with the statement that “straight white men hold far too much power in America” (adjusted prevalence difference (aPD) 6.1%, 95% CI 1.2%, 11.0%; *q* = 0.032) (Table S5). There were no significant differences in comparisons by military background on other beliefs about American society, including items assessing agreement with the QAnon ideology (Table S6).

### Non-political violence

For items measuring justification for the use of non-political violence, respondents with military backgrounds were more likely to perceive the use of force or violence as usually or always justified for self-defense (aPD 6.2%, 95% CI 2.9%, 9.5%, *q* = 0.001), and to prevent someone from injuring or killing another person (aPD 5.3%, 95% CI 1.9%, 8.7%, *q* = 0.007), relative to non-military respondents (Table S7).

### Political violence in general and to advance specific objectives

There were no significant differences by military background on items measuring the use of political violence in general and to advance 17 specific objectives with one exception—military respondents were less likely than those without a military background to view the use of political violence “to keep our borders open” as usually or always justified (aPD −3.1%, 95% CI −5.6, −0.7, *q* = 0.049) (Tables 1, Table S8).

**Table 1:** Justification for political violence, in general and for 9 specific objectives by military background

What do you think about the use of force or violence in the following situations?	Military background			No military background		
	Unweighted N	Weighted %	CI	Unweighted N	Weighted %	CI
In general what do you think about using force or violence to advance an important political objective that you support						
Never justified	1923	83.1	80.5–85.4.5.4	8773	79.6	78.5–80.6.5.6
Sometimes justified	304	15.8	13.6–18.4.6.4	1662	17.3	16.4–18.3.4.3
Usually or always justified	23	1.1	0.7–1.7.7.7	223	3.1	2.6–3.7.6.7
Adjusted prevalence difference (95% CI)	−1.19 (−2.32, −0.07)			Referent		
Thinks violence is usually or always justified to advance at least 1 of 17 objectives	929	39.0	36.2–41.8.2.8	3457	31.9	30.7–33.0.7.0
Adjusted prevalence difference (95% CI)	3.12 (−0.78, 7.03)			Referent		
To return Donald Trump to the presidency this year						
Never justified	2058	90.9	88.8–92.6.8.6	9494	88.3	87.4–89.1.4.1
Sometimes justified	79	3.9	2.8–5.4.8.4	546	6.3	5.7–7.0.7.0
Usually or always justified	94	5.2	3.9–6.9.9.9	522	5.4	4.9–6.0.9.0
Adjusted prevalence difference (95% CI)	1.13 (−1.47, 3.73)			Referent		
To stop an election from being stolen						
Never justified	1603	72.5	69.8–75.0.8.0	7913	74.9	73.8–75.9.8.9
Sometimes justified	400	17.4	15.3–19.7.3.7	1819	16.9	16.0–17.8.0.8
Usually or always justified	222	10.1	8.5–12.0.5.0	843	8.2	7.6–8.9.6.9
Adjusted prevalence difference (95% CI)	0.53 (−1.82, 2.88)			Referent		
To stop people who do not share my beliefs from voting						
Never justified	2173	95.9	94.0–97.1.0.1	10005	92.5	91.8–93.2.8.2
Sometimes justified	41	2.7	1.7–4.4.7.4	387	5.0	4.4–5.6.4.6
Usually or always justified	23	1.4	0.8–2.6.8.6	185	2.5	2.1–3.0.1.0
Adjusted prevalence difference (95% CI)	−0.17 (−1.49, 1.15)			Referent		
To prevent discrimination based on race or ethnicity						
Never justified	1466	64.2	61.4–67.0.4.0	6972	63.2	62.0–64.4.0.4
Sometimes justified	605	27.1	24.6–29.7.6.7	2783	27.6	26.5–28.7.5.7
Usually or always justified	162	8.7	7.0–10.8.0.8	812	9.2	8.5–10.0.5.0
Adjusted prevalence difference (95% CI)	2.00 (−1.33, 5.33)			Referent		
To preserve an American way of life based on Western European traditions						
Never justified	1495	68.6	65.9–71.2.9.2	7834	76.3	75.3–77.3.3.3
Sometimes justified	555	24.1	21.7–26.6.7.6	2150	18.5	17.6–19.4.6.4
Usually or always justified	172	7.3	6.0–8.8.0.8	538	5.2	4.7–5.8.7.8
Adjusted prevalence difference (95% CI)	0.77 (−1.18, 2.71)			Referent		
To preserve the American way of life l believe in						
Never justified	915	43.4	40.6–46.4.6.4	5805	57.5	56.4–58.7.4.7
Sometimes justified	919	39.6	36.8–42.4.8.4	3530	31.0	29.9–32.1.9.1
Usually or always justified	410	17.0	15.1–19.1.1.1	1287	11.5	10.7–12.2.7.2
Adjusted prevalence difference (95% CI)	0.55 (−1.90, 3.00)			Referent		
To oppose Americans who do not share my beliefs						
Never justified	2075	91.7	89.7–93.3.7.3	9671	88.9	88.1–89.7.1.7
Sometimes justified	125	6.2	4.7–8.1.7.1	746	8.2	7.5–8.9.5.9
Usually or always justified	45	2.1	1.5–3.0.5.0	218	2.9	2.5–3.4.5.4
Adjusted prevalence difference (95% CI)	0.14 (−1.41, 1.69)			Referent		
To oppose the government when it does not share my beliefs						
Never justified	1850	81.7	79.2–83.9.2.9	8757	81.4	80.4–82.4.4.4
Sometimes justified	330	15.7	13.6–18.1.6.1	1529	15.1	14.2–16.0.2.0
Usually or always justified	53	2.7	1.9–3.7.9.7	285	3.5	3.0–4.0.0.0
Adjusted prevalence difference (95% CI)	0.11 (−1.55, 1.77)			Referent		
To oppose the government when it tries to take private land for public purposes						
Never justified	1333	57.6	54.7–60.4.7.4	6537	62.0	60.8–63.2.8.2
Sometimes justified	708	32.4	29.7–35.2.7.2	3079	28.3	27.3–29.4.3.4
Usually or always justified	192	10.0	8.3–12.0.3.0	949	9.7	8.9–10.4.9.4
Adjusted prevalence difference (95% CI)	2.39 (−0.41, 5.20)			Referent		

Adjusted models include age, gender, race and ethnicity, income, education, region, political party, and potential draftee. Adjusted differences are for the usually or always justified comparison. Significance is indicated by q-values, which represent the probability that the given difference would be a false discovery, or the expected proportion of “false positives” that would be seen among the collection of all differences whose q-values were at or below the given q-value

### Personal willingness to engage in political violence

Three percent or fewer of respondents reported they were very or completely willing to personally use political violence to damage property, threaten or intimidate a person, injure a person, kill a person, or against others based on personal characteristics; there were no significant differences in comparisons by military background (Tables 2, Table S9).

**Table 2:** Personal willingness to engage in political violence based on the type of violence by military background

In a situation where you think force or violence is justified to advance an important political objective…How willing would you personally be to use force or violence in each of these ways?	Military background			No military background		
	Unweighted N	Weighted %	CI	Unweighted N	Weighted %	CI
To damage property						
Not asked the question	322	16.4	14.3–18.9.3.9	2236	22.6	21.6–23.6.6.6
Not willing	1725	73.0	70.1–75.7.1.7	7376	66.8	65.7–68.0.7.0
Somewhat willing	152	8.3	6.6–10.5.6.5	768	7.6	6.9–8.3.9.3
Very or completely willing	46	2.3	1.5–3.3.5.3	257	3.0	2.6–3.5.6.5
Adjusted prevalence difference (95% CI)	−0.05 (−1.66, 1.56)			Referent		
To threaten or intimidate a person						
Not asked the question	322	16.5	14.3–18.9.3.9	2236	22.6	21.6–23.6.6.6
Not willing	1726	73.8	71.0–76.5.0.5	7495	67.8	66.7–69.0.7.0
Somewhat willing	159	8.0	6.3–10.0.3.0	724	7.5	6.8–8.2.8.2
Very or completely willing	35	1.7	1.2–2.5.2.5	175	2.1	1.7–2.5.7.5
Adjusted prevalence difference (95% CI)	0.49 (−0.72, 1.71)			Referent		
To injure a person						
Not asked the question	322	16.5	14.3–18.9.3.9	2236	22.6	21.6–23.6.6.6
Not willing	1731	73.9	71.1–76.5.1.5	7643	69.5	68.4–70.6.4.6
Somewhat willing	146	7.8	6.2–9.8.2.8	563	5.8	5.2–6.5.2.5
Very or completely willing	41	1.8	1.3–2.6.3.6	176	2.0	1.7–2.5.7.5
Adjusted prevalence difference (95% CI)	0.07 (−1.01, 1.15)			Referent		
To kill a person						
Not asked the question	322	16.5	14.3–18.9.3.9	2236	22.6	21.6–23.6.6.6
Not willing	1781	77.0	74.3–79.4.3.4	7885	72.1	71.0–73.2.0.2
Somewhat willing	98	5.0	3.7–6.6.7.6	325	3.3	2.9–3.8.9.8
Very or completely willing	39	1.6	1.1–2.4.1.4	186	2.0	1.7–2.3.7.3
Adjusted prevalence difference (95% CI)	−0.11 (−1.04, 0.81)			Referent		

Adjusted models include age, gender, race and ethnicity, income, education, region, political party, and potential draftee. Adjusted differences are for the very or completely willing comparison. Significance is indicated by q-values, which represent the probability that the given difference would be a false discovery, or the expected proportion of “false positives” that would be seen among the collection of all differences whose q-values were at or below the given q-value

**Fig. 1:**
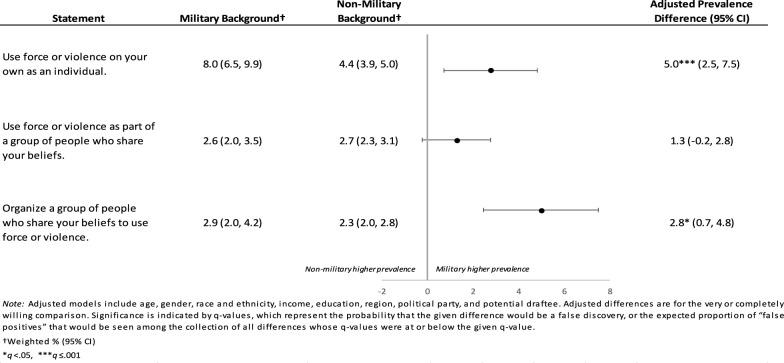
Personal Willingness to Use Political Violence as an Individual or Part of a Group by Military Background

Respondents with military backgrounds were more likely than those without to report being very or completely willing to “use force on your own as an individual” (aPD 5.0%, 95% CI 2.5%, 7.5%, *q* = 0.001) or to “organize a group of people who share your beliefs to use force or violence” (aPD 2.8%, 95% CI 0.7%, 4.8%, *q* = 0.029) to advance a political objective (Fig. 1, Table S10).

### Possession and use of firearms

**Fig. 2:**
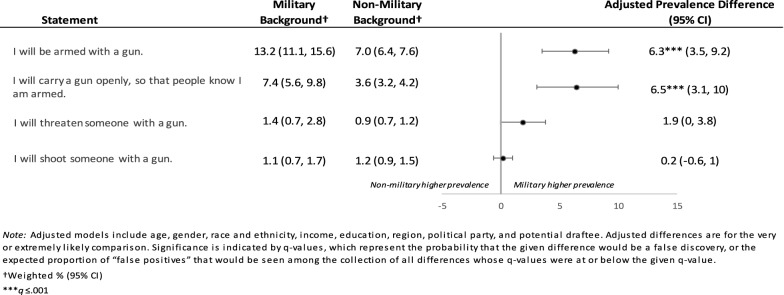
Future Likelihood of Firearm Possession and Use in a Situation Where Political Violence is Perceived as Justified by Military Background

For items measuring the use of a firearm in a situation where respondents thought force or violence was justified to advance an important political objective, respondents with a history of military service were more likely than non-military respondents to report it was very or extremely likely that “I will be armed with a gun,” (aPD 6.3%, 95% CI 3.5%, 9.2%, *q* = 0.001) and that “I will carry a gun openly, so that people know I am armed” (aPD 6.5%, 95% CI 3.1%, 10.0%, *q* = 0.001) (Fig. 2, Table S11). Respondents did not differ with respect to the likelihood of threatening or shooting someone with a gun.

### Approval of extremist right-wing groups or movements

Respondents with military backgrounds were more likely than those without a history of military service to report they strongly or very strongly approved of the Oath Keepers (aPD 4.1%, 95% CI 0.7%, 7.6%, *q* = 0.037); there were no other differences in approval of extremist right-wing groups or movements by military background (Table S12).

### Findings for combat experience

In a supplemental analysis, we examined the association between combat experience and beliefs about democracy and American society, support for political violence in different circumstances, personal willingness to engage in political violence, and approval for extremist right-wing organizations and movements among respondents with a military background and found no significant differences (Tables S13-S24).

### Associations between sociodemographics and political violence

#### Military sample

Within the sample of respondents with a military background, political party affiliation and education were associated with strong or very strong agreement with the statement “If elected leaders will not protect American democracy, the people must do it themselves, even if it requires taking violent actions”. Specifically, relative to strong Republicans, not strong Republicans (aPD −12.4%, 95% CI −21.0%, −0.04%, *q* = 0.028), those who lean Republican (aPD −9.5%, 95% CI −16.6%, −2.5%, *q* = 0.037), undecided/independent/other respondents (aPD −26.0%, 95% CI −35.3%, −16.5%, *q* = 0.001), those who lean Democrat (aPD −24.9%, 95% CI −32.0%, −17.8%, *q* = 0.001), not strong Democrats (aPD −22.5%, 95% CI −31.1%, −13.8%, *q* = 0.001), and strong Democrats (aPD −22.4%, 95% CI −30.1%, −14.8%, *q* = 0.001) were less likely to strongly or very strongly agree. In addition, respondents with some college or an associate’s degree were more likely to strongly or very strongly agree, compared to those with no high school diploma or GED (aPD 15.5%, 95% CI 5.5%, 25.4%, *q* = 0.016) (Table S25).

#### Combat sample

Due to sparse data in some demographic subgroups, we were unable to reliably estimate adjusted prevalence differences within the combat sample.

## Discussion

The current study examined variation in beliefs about democracy and American society, support for political violence, personal willingness to engage in political violence, and approval for extremist organizations and movements by military background. Few differences emerged.

Military respondents were less likely than non-military respondents to perceive the use of political violence “to keep our borders open” as usually or always justified. Respondents with a military background were more likely than respondents without a history of military service to report they were very or completely willing to “use force or violence on your own as an individual” and to “organize a group of people who share your beliefs to use force or violence.” Military respondents were also more likely than others to report that it would be very or extremely likely that they would be armed with a gun or carry a gun openly in a situation where they thought force or violence was justified to advance an important political objective; however, there were no differences with respect to threatening or shooting someone with a gun. Respondents with a military background were more likely to strongly or very strongly approve of the Oath Keepers. This difference may be in part due to the composition of the Oath Keepers, an anti-government extremist right-wing group that recruits military service members, law enforcement, and first responders under the guise of a mission to “fulfill the oath all military and police take to defend the Constitution against all enemies, foreign and domestic.”

In response to recommendations for further research into the relationship between combat experience and extremism, we conducted a supplemental analysis comparing military respondents with and without combat experience and found no differences.

Given the rarity of differences observed among numerous measures, and the relatively small size of prevalence differences (< 7%) [32], the overall picture does not suggest that military backgrounds or combat experience act as risk factors for the endorsement of political violence or extremist views, or approval of extremist right-wing organizations.

In a supplemental analysis examining variation with sociodemographic characteristics among military respondents, compared to strong Republicans, all other political groups were significantly less likely to strongly or very strongly agree that violence may be necessary if elected leaders will not protect American democracy, consistent with prior work [60]. Contrary to prior research suggesting that higher levels of education are associated with lower support for political violence [62], our analysis found that individuals with some college or an associate’s degree were significantly more likely to strongly or very strongly agree compared to those without a high school diploma or GED. However, no other education groups showed significant differences, thus the direction of the association was not consistently inverse with increasing educational attainment. Future research should examine sociodemographic variation among individuals with military and combat backgrounds more broadly in order to identity subgroups within these populations who may be at elevated risk of supporting or being willing to engage in various forms of political violence and to inform tailored prevention efforts among active-duty service members, those with combat experience, and veterans.

Our findings in the primary analysis are perhaps unexpected, given extant research indicating that individuals with military backgrounds are overrepresented among offenders involved in crimes or plots motivated by extremist ideologies. However, samples of extremist offenders are not representative of the general population. For example, whereas 50% of the U.S. population is female, and 59% is non-Hispanic white [56], 89% of individuals charged, arrested, or indicted with committing extremist crimes between 1948 and 2021 were male, and of those whose race and ethnicity were known, 72% were white, non-Hispanic [41]. Notably, the proportion of white, adult males who are veterans is twice that of the general population—14% [55].

Further, results are consistent with one prior study indicating approval of extremist views, political violence, and extremist groups and movements among veterans was not higher than that observed in the general population. In a report published by the RAND Corporation, Helmus et al. [22] found that similar proportions of veterans and respondents from the general population mostly or completely agreed that “because things have gotten so far off track, true American patriots may have to resort to violence in order to save our country.” Our results on the same item were consistent.

Whereas Helmus et al. [22] found a somewhat higher proportion of the general population than veterans supported the Proud Boys, white supremacists, and statements endorsing QAnon and the Great Replacement theory, we did not find differences in strong or very strong approval of white supremacism or the Proud Boys or strong or very strong agreement with two QAnon items, one of which was identical to the item [22] used, or with the statement “In America, native-born white people are being replaced by immigrants”. Our questions regarding approval of extremist groups or movements did differ from those of [22], who asked about opinions (e.g. What is your opinion of Proud Boys?), with response options ranging from “Very unfavorable” to “Very favorable”, as well as the option “Never heard of”. In contrast with the current study, [22] did not conduct significance tests, and they included those who responded “somewhat favorable” in their results on extremist groups or movements, which may account in part for the differences in our conclusions on these items.

In a departure from our results, Pape et al. [43] found that veterans were nearly twice as likely as non-veterans to endorse high insurrectionist statements. This may be due to the differences in the analytic approach and the use of survey weights, the composition of the military sample (veterans only versus veterans and active-duty respondents), and the age range of respondents (under 65 versus the inclusion of 65 +).

Variability among findings in prior studies and the current study underscores the importance of future exploration of support for and willingness to engage in political violence in military populations relative to those without military experience with a diversity of political violence measures and analytic approaches.

Irrespective of our results, it should be noted that some experts have identified military service as a potential risk factor for ideologically motivated violence and extremism as a possibly growing problem in the military community, particularly with respect to veterans [29, 52, 64]. Our findings may reflect that population-based surveys are insensitive to rare signals that are difficult to detect. Perhaps extremists in the military are more likely to participate in violence or become offenders than extremists in the general population. Given the expertise and experience veterans and service members provide to extremist groups or movements, even if individuals with military backgrounds are not at heightened risk for extremist attitudes, the risks presented by their involvement in political violence should not be understated [22].

## Limitations

Findings must be considered alongside several limitations. The findings are cross-sectional and subject to sampling error and nonresponse bias. Many outcomes are uncommon, with response counts < 100 and weighted prevalences below 5%. The large study sample and small prevalence estimates result in relatively narrow confidence intervals in these cases, but the estimates remain vulnerable to bias from sources such as inattentive or strategic responses. Findings on support for political violence to advance 1 or more of 17 political objectives are reflective of the specific objectives presented; different items may have resulted in different outcomes. Widely publicized mass shootings occurred in Buffalo, NY and Uvalde, TX while the survey was in the field. The Buffalo shooting is understood to have been a race-related hate crime motivated by great replacement thinking and may have affected respondents’ views on race, violence, and that particular belief. Russia’s war against Ukraine may have influenced responses on violence and democracy.

While we did not measure military branch affiliations, prior research indicates disproportionate representation of the Marine Corps branch among criminal extremists with military backgrounds [29]. We cannot discern whether heightened scrutiny on extremism in the military, particularly after the January 6th insurrection, may have influenced socially desirable responding among respondents with military backgrounds. Even among respondents without military backgrounds, the sensitivity of and stigma associated with the topic of political violence may have introduced social desirability bias. We did not ask respondents to report prior involvement in political violence or extremist groups or movements.

Regarding our analysis on sociodemographic variations in agreement with the potential need for violence in the United States, limited sample sizes within certain demographic subgroups of the combat sample reduced our ability to generate stable adjusted estimates. Future studies with larger samples of this population may be better positioned to explore within-group variation. In addition, we only examined one outcome, which limits conclusions.

## Conclusion

Findings from our study provide room for optimism; they largely do not support concerns that military service or combat experience act as risk factors for the endorsement of political violence, agreement with extremist views, or approval of extremist groups or movements. However, our study was designed to detect population-level signals; survey methodologies are insensitive to rare occurrences among small groups of people. In addition to the need for continued exploration of the prevalence of extremist attitudes among veterans and active-duty service members, future research should explore the mechanisms underlying radicalization among individuals with a military background or combat experience; prior research suggests extremist offenders with military backgrounds are overwhelmingly veterans, indicating separation from the military may be a critical stage of intervention [28]. Likewise, as previous work indicates that there appears to be differential risk of involvement in political violence based on branch affiliation [29], experts have recommended that future research should explore these differences and how they may relate to branch cultures and subcultures [22].

## Supplementary Information

Supplementary materials 1

## Data Availability

No datasets were generated or analysed during the current study.

## Abbreviations

CI: Confidence interval
aPD: Adjusted prevalence difference
