# Supplementary material for: Militancy in the military: military service and support for political violence and right-wing extremism
Source: Inj Epidemiol. 2025 Nov 26;12:80. doi: 10.1186/s40621-025-00626-5 (PMC12659064; doi:10.1186/s40621-025-00626-5)
Supplement: Supplementary file 1 [file 40621_2025_626_MOESM1_ESM.docx]

Less than $10,000	33	1.5	1.0-2.3	338	4.2	3.7-4.7
$10,000 to $24,999	138	7.8	6.2-9.6	940	9.1	8.4-9.8
$25,000 to $49,999	383	16.9	14.7-19.3	1849	17.0	16.1-18.0
$50,000 to $74,999	448	17.9	15.8-20.1	1788	16.2	15.3-17.1
$75,000 to $99,999	419	15.7	13.9-17.6	1580	13.0	12.2-13.7
$100,000 to $149,999	475	20.4	18.2-22.8	1935	17.6	16.7-18.5
$150,000 or more	359	19.9	17.6-22.4	2262	23.0	22.0-24.0
Political party						
Strong Republican	574	23.4	21.2-25.7	1990	14.8	14.1-15.6
Not Strong Republican	248	11.2	9.5-13.2	1214	10.6	9.9-11.4
Leans Republican	517	22.1	19.8-24.6	1761	15.5	14.7-16.4
Undecided/Independent/Other	72	3.3	2.5-4.3	344	3.9	3.5-4.5
Leans Democrat	354	16.8	14.6-19.2	2002	21.7	20.6-22.7
Not Strong Democrat	170	9.1	7.5-11.1	1340	14.8	13.9-15.7
Strong Democrat	317	14.2	12.3-16.4	1999	18.6	17.7-19.6
Draft						
Not potential draftee	1117	63.6	61.03-66.14	10692	100.0	--
Potential draftee	1138	36.4	33.86-38.97	0	0.0	--

Some college or Associate's degree	422	39.3	35.5-43.3	460	39.6	36.0-43.3
Bachelor's degree	300	21.6	19.0-24.5	255	17.6	15.2-20.3
Master's degree or higher	264	20.9	18.2-23.8	198	14.4	12.3-16.8
Region						
Northeast	124	10.4	8.2-13.0	168	17.2	14.4-20.5
Midwest	252	21.0	18.3-24.0	208	16.9	14.3-19.8
South	464	45.0	41.0-49.0	475	43.5	39.8-47.3
West	265	23.7	19.8-27.9	299	22.4	19.5-25.6
Income						
Less than $10,000	19	1.5	0.9-2.4	14	1.5	0.8-2.7
$10,000 to $24,999	49	6.0	3.9-9.2	89	8.6	6.7-11.1
$25,000 to $49,999	192	17.5	14.1-21.4	191	16.6	13.9-19.7
$50,000 to $74,999	234	19.9	17.1-22.9	214	16.9	14.3-19.9
$75,000 to $99,999	234	18.2	15.7-21.1	185	14.5	12.2-17.1
$100,000 to $149,999	238	21.8	18.7-25.2	237	19.8	16.9-23.0
$150,000 or more	139	15.2	12.3-18.5	220	22.1	19.1-25.5
Political party						
Strong Republican	276	24.8	21.7-28.3	298	22.7	19.9-25.7
Not Strong Republican	116	10.3	8.2-12.9	132	11.6	9.3-14.3
Leans Republican	270	24.0	20.7-27.7	247	21.2	18.2-24.6
Undecided/Independent/Other	36	3.1	2.2-4.5	36	3.3	2.3-4.8
Leans Democrat	170	14.4	12.1-17.1	184	17.9	15.0-21.2
Not Strong Democrat	82	10.0	7.1-14.0	88	8.7	6.8-11.0
Strong Democrat	154	13.3	10.7-16.3	163	14.7	12.2-17.6
Draft						
Not potential draftee	501	59.7	56.0-63.3	616	65.5	62.1-68.7
Potential draftee	604	40.3	36.7-44.0	534	34.5	31.3-37.9

Adjusted prevalence difference (95% CI)	0.93 (-1.33, 3.19)			Referent		
These days, American democracy only serves the interests of the wealthy and powerful.						
Do not agree	873	34.1	31.6-36.7	3103	25.9	24.9-26.9
Somewhat agree	739	34.8	32.1-37.6	3760	36.8	35.7-38.0
Strongly or very strongly agree	636	31.1	28.4-34.0	3718	37.3	36.2-38.5
Adjusted prevalence difference (95% CI)	-0.21 (-4.36, 3.95)			Referent		
Having a strong leader for America is more important than having a democracy.						
Do not agree	1518	63.3	60.3-66.1	6403	56.5	55.3-57.7
Somewhat agree	362	18.4	16.1-21.0	2266	24.0	22.9-25.0
Strongly or very strongly agree	359	18.3	16.1-20.8	1895	19.5	18.5-20.5
Adjusted prevalence difference (95% CI)	-0.18 (-3.67, 3.31)			Referent		
The 2020 election was stolen from Donald Trump, and Joe Biden is an illegitimate president.						
Do not agree	1364	62.3	59.5-65.1	7078	68.6	67.5-69.7
Somewhat agree	381	15.7	13.8-17.8	1449	13.6	12.7-14.4
Strongly or very strongly agree	482	22.0	19.7-24.4	2020	17.8	16.9-18.7
Adjusted prevalence difference (95% CI)	1.22 (-1.36, 3.80)			Referent		
Armed citizens should patrol polling places at election time.						
Do not agree	1978	87.3	85.2-89.1	9065	83.8	82.8-84.7
Somewhat agree	162	8.0	6.5-9.7	888	9.7	9.0-10.5
Strongly or very strongly agree	94	4.7	3.7-6.1	599	6.5	5.9-7.2
Adjusted prevalence difference (95% CI)	-0.05 (-2.16, 2.07)			Referent		

Note: Adjusted models include age, gender, race and ethnicity, income, education, region, political party, and potential draftee. Adjusted differences are for the first response option comparison for the initial four statements and for the strongly or very strongly agree comparison for the remaining statements. Significance is indicated by q-values, which represent the probability that the given difference would be a false discovery, or the expected proportion of "false positives" that would be seen among the collection of all differences whose q-values were at or below the given q-value.

Table S4. Beliefs Concerning the Potential Need for Violence in the USA By Military Background.

Statement	Military background			No military background		
	Unweighted N	Weighted %	CI	Unweighted N	Weighted %	CI
If elected leaders will not protect American democracy, the people must do it themselves, even if it requires taking violent actions.						
Do not agree	1016	45.6	42.7-48.5	5445	51.7	50.4-52.9
Somewhat agree	692	31.8	29.2-34.5	3146	29.9	28.8-31.1
Strongly or very strongly agree	530	22.7	20.4-25.1	1972	18.4	17.5-19.4
Adjusted prevalence difference (95% CI)	2.49 (-0.83, 5.81)			Referent		
Our American way of life is disappearing so fast that we may have to use force to save it.						
Do not agree	1278	55.9	53.0-58.7	6082	57.0	55.7-58.1
Somewhat agree	611	27.8	25.2-30.5	2795	27.0	26.0-28.1
Strongly or very strongly agree	346	16.4	14.4-18.6	1686	16.0	15.1-16.9
Adjusted prevalence difference (95% CI)	0.47 (-2.54, 3.49)			Referent		
Because things have gotten so far off track, true American patriots may have to resort to violence in order to save our country.						
Do not agree	1609	70.5	67.6-73.1	7877	74.4	73.3-75.5
Somewhat agree	453	21.5	19.0-24.1	1834	17.8	16.8-18.7
Strongly or very strongly agree	169	8.1	6.6-9.8	823	7.8	7.2-8.5
Adjusted prevalence difference (95% CI)	0.96 (-1.46, 3.39)			Referent		
In the next few years there will be civil war in the United States.						
Do not agree	1200	50.1	47.2-53.0	5207	48.5	47.3-49.7
Somewhat agree	773	36.5	33.7-39.4	3973	37.5	36.4-38.7
Strongly or very strongly agree	260	13.4	11.4-15.7	1344	14.0	13.2-14.9
Adjusted prevalence difference (95% CI)	4.04 (0.45, 7.64)			Referent		

Note: Adjusted models include age, gender, race and ethnicity, income, education, region, political party, and potential draftee. Adjusted differences are for the strongly or very strongly agree comparison. Significance is indicated by q-values, which represent the probability that the given difference would be a false discovery, or the expected proportion of "false positives" that would be seen [illegible]

Strongly or very strongly agree	437	24.2	21.5-27.1	3086	33.4	32.3-34.6
Adjusted prevalence difference (95% CI)	6.11* (1.23, 10.99)			Referent		
Discrimination against whites is as big a problem as discrimination against Blacks and other minorities.						
Do not agree	902	41.7	38.9-44.6	5105	51.0	49.8-52.2
Somewhat agree	588	25.2	22.8-27.8	2483	22.7	21.7-23.7
Strongly or very strongly agree	752	33.1	30.5-35.8	3007	26.3	25.2-27.3
Adjusted prevalence difference (95% CI)	2.25 (-0.72, 5.23)			Referent		
Having more Black Americans, Latinos, and Asian Americans is good for the country.						
Do not agree	569	23.7	21.3-26.1	2205	18.4	17.5-19.3
Somewhat agree	863	37.2	34.5-40.0	3732	34.8	33.7-36.0
Strongly or very strongly agree	785	39.2	36.3-42.1	4535	46.8	45.6-48.0
Adjusted prevalence difference (95% CI)	4.69 (0.12, 9.25)			Referent		
In America, native-born white people are being replaced by immigrants.						
Do not agree	1130	52.6	49.8-55.5	6006	59.4	58.2-60.5
Somewhat agree	715	30.6	28.0-33.2	2768	24.8	23.8-25.9
Strongly or very strongly agree	393	16.8	14.9-18.9	1797	15.8	15.0-16.7
Adjusted prevalence difference (95% CI)	-1.58 (-4.16, 0.99)			Referent		

Note: Adjusted models include age, gender, race and ethnicity, income, education, region, political party, and potential draftee. Adjusted differences are for the strongly or very strongly agree comparison. Significance is indicated by q-values, which represent the probability that the given difference would be a false discovery, or the expected proportion of "false positives" that would be seen among the collection of all differences whose q-values were at or below the given q-value.

*q<.05

Table S6. Beliefs Concerning Qanon and the Biblical "End Times" by Military Background.

Statement	Military background			No military background		
	Unweighted N	Weighted %	CI	Unweighted N	Weighted %	CI
The government, media, and financial worlds in the U.S. are controlled by a group of Satan-worshipping pedophiles who run a global child sex trafficking operation.						
Do not agree	1864	80.0	77.3-82.4	8412	76.9	75.7-77.9
Somewhat agree	222	11.8	9.8-14.1	1258	14.1	13.2-15.0
Strongly or very strongly agree	137	8.2	6.7-10.1	816	9.1	8.3-9.8
Adjusted prevalence difference (95% CI)	1.95 (-0.88, 4.78)			Referent		
There is a storm coming soon that will sweep away the elites in power and restore the rightful leaders.						
Do not agree	1596	69.9	67.0-72.5	7468	69.9	68.8-71.0
Somewhat agree	438	20.2	17.9-22.8	2036	20.0	19.0-20.9
Strongly or very strongly agree	191	9.9	8.2-11.9	971	10.1	9.4-10.9
Adjusted prevalence difference (95% CI)	0.03 (-2.47, 2.53)			Referent		
The chaos in America today is evidence that we are living in what the Bible calls "the end times."						
Do not agree	1347	57.1	54.2-60.0	6065	55.6	54.4-56.8
Somewhat agree	556	25.6	23.1-28.3	2581	24.8	23.8-25.9
Strongly or very strongly agree	330	17.3	15.1-19.6	1895	19.6	18.6-20.6
Adjusted prevalence difference (95% CI)	-0.35 (-3.77, 3.07)			Referent		

Note: Adjusted models include age, gender, race and ethnicity, income, education, region, political party, and potential draftee. Adjusted differences are for the strongly or very strongly agree comparison. Significance is indicated by q-values, which represent the probability that the given difference would be a false discovery, or the expected proportion of "false positives" that would be seen among the collection of all differences whose q-values were at or below the given q-value.

Table S7. Justification for Violence in Non-Political Situations by Military Background.

What do you think about the use of force or violence in the following situations?	Military background			No military background		
	Unweighted N	Weighted %	CI	Unweighted N	Weighted %	CI
In self defense						
Never justified	16	0.8	0.4-1.6	191	2.2	1.9-2.7
Sometimes justified	298	13.8	11.8-16.0	2180	22.9	21.9-24.0
Usually or always justified	1935	85.4	83.1-87.4	8255	74.8	73.8-75.9
Adjusted prevalence difference (95% CI)	6.19*** (2.92, 9.46)			Referent		
To prevent someone from injuring or killing another person						
Never justified	15	0.9	0.5-1.7	192	2.4	2.0-2.8
Sometimes justified	222	11.3	9.4-13.6	1936	21.0	20.0-22.1
Usually or always justified	1999	87.8	85.5-89.8	8466	76.6	75.5-77.6
Adjusted prevalence difference (95% CI)	5.31** (1.94, 8.68)			Referent		
To prevent someone from injuring or killing themselves						
Never justified	116	6.5	5.0-8.3	607	6.2	5.6-6.9
Sometimes justified	696	31.1	28.4-33.9	3598	35.2	34.0-36.3
Usually or always justified	1430	62.5	59.6-65.3	6396	58.6	57.4-59.8
Adjusted prevalence difference (95% CI)	-0.51 (-4.86, 3.84)			Referent		
To prevent harm or damage to property						
Never justified	241	11.8	10.0-13.8	1619	15.9	15.0-16.8
Sometimes justified	1083	48.1	45.3-51.0	5307	50.5	49.3-51.7
Usually or always justified	921	40.1	37.3-42.9	3674	33.6	32.5-34.7
Adjusted prevalence difference (95% CI)	-0.50 (-4.31, 3.32)			Referent		
To win an argument						
Never justified	2037	89.3	87.0-91.2	9396	86.4	85.5-87.3
Sometimes justified	161	7.7	6.1-9.6	884	9.6	8.9-10.4
Usually or always justified	43	3.1	2.1-4.6	327	4.0	3.5-4.5
Adjusted prevalence difference (95% CI)	0.89 (-1.48, 3.27)			Referent		
In response to an insult						
Never justified	1999	86.8	84.4-88.9	9071	82.1	81.1-83.1
Sometimes justified	191	9.7	7.9-11.9	1151	13.4	12.5-14.3
Usually or always justified	53	3.5	2.4-5.0	385	4.5	4.0-5.1
Adjusted prevalence difference (95% CI)	0.31 (-2.05, 2.67)			Referent		
To get respect						
Never justified	2078	90.3	88.1-92.2	9512	86.8	85.8-87.6
Sometimes justified	114	6.5	5.0-8.4	721	8.4	7.7-9.1
Usually or always justified	52	3.2	2.2-4.7	376	4.9	4.3-5.5
Adjusted prevalence difference (95% CI)	0.18 (-2.31, 2.67)			Referent		

Note: Adjusted models include age, gender, race and ethnicity, income, education, region, political party, and potential draftee. Adjusted differences are for the usually or always justified comparison. Significance is indicated by q-values, which represent the probability that the given difference would be a false discovery, or the expected proportion of "false positives" that would be seen among the collection of all differences whose q-values were at or below the given q-value.

Table S8. Justification for Political Violence for 8 Additional Specific Objectives by Military Background (these objectives were paired, with respondents randomized 1:1 to see 1 item in each pair).

What do you think about the use of force or violence in the following situations?	Military background			No military background		
	Unweighted N	Weighted %	CI	Unweighted N	Weighted %	CI
To stop voter fraud						
Never justified	798	70.0	66.1-73.6	3974	74.5	72.9-75.9
Sometimes justified	199	17.2	14.3-20.5	824	16.4	15.2-17.7
Usually or always justified	136	12.8	10.2-15.9	488	9.1	8.2-10.2
Adjusted prevalence difference (95% CI)	1.52 (-2.55, 5.60)			Referent		
To stop voter intimidation						
Never justified	585	58.0	54.0-61.9	3262	62.0	60.3-63.6
Sometimes justified	375	30.5	27.0-34.2	1528	27.8	26.3-29.3
Usually or always justified	152	11.6	9.4-14.1	553	10.2	9.2-11.3
Adjusted prevalence difference (95% CI)	-0.91 (-4.14, 2.31)			Referent		
To reinforce the police						
Never justified	296	29.7	26.1-33.6	2081	44.0	42.3-45.7
Sometimes justified	531	43.0	39.1-46.9	2130	38.6	36.9-40.2
Usually or always justified	348	27.3	24.2-30.7	1056	17.5	16.3-18.7
Adjusted prevalence difference (95% CI)	2.27 (-1.49, 6.04)			Referent		
To stop police violence						
Never justified	551	48.5	44.2-52.7	2563	45.6	43.9-47.3
Sometimes justified	409	40.7	36.5-45.0	2171	41.4	39.8-43.1
Usually or always justified	107	10.9	8.5-13.8	624	13.0	11.9-14.2
Adjusted prevalence difference (95% CI)	-0.60 (-4.93, 3.73)			Referent		
To stop illegal immigration						
Never justified	523	51.3	47.2-55.4	3210	62.6	61.0-64.2
Sometimes justified	369	33.3	29.5-37.3	1450	26.1	24.6-27.6
Usually or always justified	181	15.4	12.7-18.5	677	11.3	10.3-12.4
Adjusted prevalence difference (95% CI)	-1.95 (-5.41, 1.50)			Referent		
To keep our borders open						
Never justified	838	72.9	69.3-76.3	3563	66.0	64.4-67.6
Sometimes justified	234	20.1	17.0-23.5	1301	25.6	24.2-27.2
Usually or always justified	98	7.0	5.5-8.9	420	8.4	7.4-9.4
Adjusted prevalence difference (95% CI)	-3.14* (-5.55, -0.73)			Referent		
To stop a protest or demonstration						
Never justified	625	54.9	51.0-58.7	3057	58.7	57.0-60.3
Sometimes justified	475	40.3	36.5-44.2	1921	35.1	33.5-36.7
Usually or always justified	64	4.9	3.6-6.5	312	6.2	5.4-7.2
Adjusted prevalence difference (95% CI)	-0.92 (-3.35, 1.51)			Referent		
To support a protest or demonstration						
Never justified	914	80.9	76.9-84.4	4330	78.7	77.3-80.1
Sometimes justified	135	15.4	12.1-19.2	800	16.7	15.4-18.0
Usually or always justified	33	3.7	2.4-5.7	213	4.6	4.0-5.4
Adjusted prevalence difference (95% CI)	-1.57 (-3.49, 0.36)			Referent		

Note: Adjusted models include age, gender, race and ethnicity, income, education, region, political party, and potential draftee. Adjusted differences are for the usually or always justified comparison. Significance is indicated by q-values, which represent the probability that the given difference would be a false discovery, or the expected proportion of "false positives" that would be seen among the collection of all differences whose q-values were at or below the given q-value.

Table S9. Personal Willingness to Engage in Political Violence Based on the Target of Violence by Military Background.

In a situation where you think force or violence is justified to advance an important political objective…How willing would you personally be to use force or violence against a person because they are…	Military background			No military background		
	Unweighted N	Weighted %	CI	Unweighted N	Weighted %	CI
An elected federal or state government official						
Not asked the question	322	16.6	14.4-19.0	2236	22.7	21.7-2[illegible]
Not willing	1755	75.9	73.1-78.5	7754	70.8	69.7-7[illegible]
Somewhat willing	130	6.2	4.7-8.0	452	4.5	4.0-5.[illegible]
Very or completely willing	28	1.4	0.8-2.5	158	2.0	1.6-2.[illegible]
Adjusted prevalence difference (95% CI)	0.16 (-1.21, 1.53)			Referent		
An elected local government official						
Not asked the question	322	16.5	14.3-19.0	2236	22.7	21.7-2[illegible]
Not willing	1782	76.8	74.0-79.3	7800	71.5	70.4-7[illegible]
Somewhat willing	109	5.5	4.1-7.4	406	4.1	3.6-4.[illegible]
Very or completely willing	24	1.2	0.7-2.1	144	1.7	1.4-2.[illegible]
Adjusted prevalence difference (95% CI)	0.13 (-1.20, 1.47)			Referent		
An election worker such as a poll worker or vote counter						
Not asked the question	322	16.5	14.3-18.9	2236	22.7	21.7-2[illegible]
Not willing	1849	79.7	77.0-82.1	8025	73.1	72.0-7[illegible]
Somewhat willing	53	3.0	1.9-4.7	230	2.7	2.3-3.[illegible]
Very or completely willing	18	0.9	0.5-1.4	113	1.6	1.3-2.[illegible]
Adjusted prevalence difference (95% CI)	-0.05 (-1.06, 0.96)			Referent		
A public health official						
Not asked the question	322	16.6	14.4-19.0	2236	22.7	21.7-2[illegible]
Not willing	1822	78.6	75.9-81.0	7928	72.4	71.3-7[illegible]
Somewhat willing	74	3.5	2.5-5.0	312	3.4	3.0-3.[illegible]
Very or completely willing	21	1.4	0.7-2.6	116	1.5	1.2-1.[illegible]
Adjusted prevalence difference (95% CI)	1.13 (-0.76, 3.02)			Referent		
A member of the military or National Guard						
Not asked the question	322	16.5	14.3-19.0	2236	22.7	21.7-2[illegible]
Not willing	1800	76.5	73.7-79.1	7851	71.5	70.4-7[illegible]
Somewhat willing	83	4.5	3.3-6.1	367	4.0	3.5-4.[illegible]
Very or completely willing	34	2.5	1.5-4.1	146	1.9	1.5-2.[illegible]
Adjusted prevalence difference (95% CI)	3.51 (0.58, 6.45)			Referent		
A police officer						
Not asked the question	322	16.5	14.4-19.0	2236	22.7	21.7-2[illegible]
Not willing	1796	77.3	74.5-79.8	7753	70.4	69.3-7[illegible]
Somewhat willing	91	4.6	3.3-6.3	440	4.7	4.2-5.[illegible]
Very or completely willing	28	1.7	1.0-2.6	176	2.3	1.9-2.[illegible]
Adjusted prevalence difference (95% CI)	0.94 (-0.88, 2.76)			Referent		
A person who does not share your race or ethnicity						
Not asked the question	322	16.5	14.3-18.9	2236	22.7	21.7-2[illegible]
Not willing	1853	79.4	76.7-81.8	8012	73.0	71.9-7[illegible]
Somewhat willing	48	2.8	1.8-4.3	242	2.9	2.4-3.[illegible]
Very or completely willing	19	1.4	0.7-2.7	107	1.5	1.2-1.[illegible]
Adjusted prevalence difference (95% CI)	1.45 (-0.84, 3.74)			Referent		
A person who does not share your religion						
Not asked the question	322	16.5	14.3-19.0	2236	22.7	21.7-2[illegible]
Not willing	1864	79.8	77.1-82.3	8033	73.3	72.2-7[illegible]
Somewhat willing	35	2.6	1.5-4.2	220	2.7	2.2-3.[illegible]
Very or completely willing	16	1.1	0.5-2.3	101	1.3	1.1-1.[illegible]
Adjusted prevalence difference (95% CI)	0.97 (-0.61, 2.56)			Referent		
A person who does not share your political beliefs						
Not asked the question	322	16.5	14.3-19.0	2236	22.7	21.7-2[illegible]
Not willing	1826	78.7	76.0-81.1	7931	72.4	71.3-7[illegible]
Somewhat willing	75	3.9	2.8-5.4	328	3.6	3.1-4.[illegible]
Very or completely willing	16	1.0	0.5-1.9	103	1.4	1.1-1.[illegible]
Adjusted prevalence difference (95% CI)	0.52 (-0.95, 1.99)			Referent		

Note: Adjusted models include age, gender, race and ethnicity, income, education, region, political party, and potential draftee. Adjusted differences are for the very or completely willing comparison. Significance is indicated by q-values, which represent the probability that the given difference would be a false discovery, or the expected [illegible]

Table S10. Personal Willingness to Use Political Violence as an Individual or Part of a Group by Military Background.

You agreed that the use of force or violence could be justified to advance one/some of the political objectives we just discussed. In that/those situation/situations, how willing would you personally be to…	Military background			No military background		
	Unweighted N	Weighted %	CI	Unweighted N	Weighted %	CI
Use force or violence on your own as an individual						
Not asked the question	322	16.5	14.3-18.9	2236	22.6	21.6-2[illegible]
Not willing	1302	55.5	52.5-58.4	6232	57.5	56.3-5[illegible]
Somewhat willing	463	20.0	17.8-22.5	1695	15.5	14.6-1[illegible]
Very or completely willing	156	8.0	6.5-9.9	465	4.4	3.9-5.[illegible]
Adjusted prevalence difference (95% CI; q-value)	4.99*** (2.46, 7.51)			Referent		
Use force or violence as part of a group of people who share your beliefs						
Not asked the question	322	16.5	14.3-18.9	2236	22.6	21.6-2[illegible]
Not willing	1521	64.9	62.0-67.7	6968	64.0	62.8-6[illegible]
Somewhat willing	333	16.0	13.8-18.4	1177	10.7	10.0-1[illegible]
Very or completely willing	65	2.6	2.0-3.5	241	2.7	2.3-3.[illegible]
Adjusted prevalence difference (95% CI; q-value)	1.28 (-0.22, 2.78)			Referent		
Organize a group of people who share your beliefs to use force or violence						
Not asked the question	322	16.5	14.3-18.9	2236	22.6	21.6-2[illegible]
Not willing	1671	72.0	69.1-74.6	7460	67.7	66.5-6[illegible]
Somewhat willing	196	8.7	7.1-10.5	740	7.4	6.7-8.[illegible]
Very or completely willing	54	2.9	2.0-4.2	197	2.3	2.0-2.[illegible]
Adjusted prevalence difference (95% CI; q-value)	2.78* (0.73, 4.83)			Referent		

Note: Adjusted models include age, gender, race and ethnicity, income, education, region, political party, and potential draftee. Adjusted differences are for the very or completely willing comparison. Significance is indicated by q-values, which represent the probability that the given difference would be a false discovery, or the expected [illegible]

Thinking now about the future and all the changes it might bring, how likely is it that you will use a gun in any of the following ways in the next few years—in a situation where you think force or violence is justified to advance an important political objective?	Military background			No military background		
	Unweighted N	Weighted %	CI	Unweighted N	Weighted %	CI
I will be armed with a gun.						
Not likely	1760	76.9	74.1-79.4	8648	82.3	81.3-83.2
Somewhat likely	224	10.0	8.4-11.8	1107	10.8	10.0-11.6
Very or extremely likely	245	13.2	11.1-15.6	825	7.0	6.4-7.6
Adjusted prevalence difference (95% CI)	6.34*** (3.53, 9.15)			Referent		
I will carry a gun openly, so that people know I am armed.						
Not likely	1963	85.9	83.4-88.1	9596	90.8	90.0-91.5
Somewhat likely	156	6.7	5.5-8.2	595	5.6	5.0-6.2
Very or extremely likely	107	7.4	5.6-9.8	382	3.6	3.2-4.1
Adjusted prevalence difference (95% CI)	6.53*** (3.10, 9.95)			Referent		
I will threaten someone with a gun.						
Not likely	2183	97.1	95.4-98.1	10387	97.8	97.4-98.2
Somewhat likely	27	1.5	0.8-2.9	121	1.3	1.0-1.6
Very or extremely likely	18	1.4	0.7-2.8	65	0.9	0.7-1.2
Adjusted prevalence difference (95% CI)	1.90 (0.03, 3.78)			Referent		
I will shoot someone with a gun.						
Not likely	2142	95.8	94.3-96.9	10230	96.3	95.8-96.8
Somewhat likely	62	3.1	2.1-4.6	240	2.5	2.1-3.0
Very or extremely likely	26	1.1	0.7-1.7	106	1.2	0.9-1.5
Adjusted prevalence difference (95% CI)	0.17 (-0.63, 0.97)			Referent		

Note: Adjusted models include age, gender, race and ethnicity, income, education, region, political party, and potential draftee. Adjusted differences are for the very or extremely likely comparison. Significance is indicated by q-values, which represent the probability that the given difference would be a false discovery, or the expected proportion of "false positives" that would be seen among the collection of all differences whose q-values were at or below the given q-value.

***$q \leq .001$

Table S12. Approval of Extremist Organizations and Movements by Military Background.

How much do you approve or disapprove of these named groups and organizations?	Military background			No military background		
	Unweighted N	Weighted %	CI	Unweighted N	Weighted %	CI
Proud Boys						
Do not approve	1365	60.0	57.2-62.8	6004	53.8	52.6-55.0
Somewhat approve	118	5.1	4.1-6.5	397	3.8	3.4-4.3
Strongly or very strongly approve	42	2.3	1.5-3.3	190	2.2	1.8-2.6
I don't know enough to rate it or I have never heard about it	719	32.6	29.9-35.4	3994	40.2	39.1-41.4
Adjusted prevalence difference (95% CI)	0.80 (-1.35, 2.96)			Referent		
Oath Keepers						
Do not approve	1073	45.6	42.8-48.5	4247	35.9	34.8-37.0
Somewhat approve	121	4.8	3.8-5.9	392	3.6	3.1-4.1
Strongly or very strongly approve	90	4.2	3.2-5.4	242	2.6	2.2-3.1
I don't know enough to rate it or I have never heard about it	958	45.5	42.6-48.4	5696	57.9	56.8-59.1
Adjusted prevalence difference (95% CI)	4.14* (0.71, 7.57)			Referent		
Three Percenters						
Do not approve	775	34.2	31.5-36.9	3028	25.9	24.9-26.9
Somewhat approve	56	2.5	1.8-3.4	237	2.5	2.1-2.9
Strongly or very strongly approve	31	1.7	1.1-2.8	135	1.7	1.4-2.1
I don't know enough to rate it or I have never heard about it	1379	61.6	58.8-64.4	7176	69.9	68.8-71.0
Adjusted prevalence difference (95% CI)	2.29 (-1.97, 6.56)			Referent		
Militia movement						
Do not approve	1241	53.5	50.5-56.3	5295	45.8	44.6-47.0
Somewhat approve	143	6.4	5.1-8.0	501	4.5	4.0-5.1
Strongly or very strongly approve	55	2.9	2.0-4.1	180	2.1	1.7-2.5
I don't know enough to rate it or I have never heard about it	801	37.3	34.4-40.2	4587	47.6	46.4-48.8
Adjusted prevalence difference (95% CI)	3.26 (0.36, 6.17)			Referent		
White supremacy movement						
Do not approve	1870	83.4	81.2-85.4	8549	77.4	76.3-78.5
Somewhat approve	37	2.2	1.5-3.2	187	2.3	1.9-2.7
Strongly or very strongly approve	16	0.9	0.4-1.7	99	1.2	0.9-1.5
I don't know enough to rate it or I have never heard about it	318	13.5	11.8-15.5	1722	19.1	18.2-20.2
Adjusted prevalence difference (95% CI)	0.40 (-1.16, 1.96)			Referent		
Christian nationalist movement						
Do not approve	880	38.8	36.0-41.6	4112	36.7	35.6-37.9
Somewhat approve	191	8.4	7.0-10.1	698	6.4	5.8-7.0
Strongly or very strongly approve	96	5.1	3.9-6.7	445	4.7	4.2-5.3
I don't know enough to rate it or I have never heard about it	1072	47.7	44.8-50.6	5304	52.2	51.0-53.4
Adjusted prevalence difference (95% CI)	0.23 (-3.19, 3.66)			Referent		
Boogaloo movement						
Do not approve	824	36.5	33.8-39.2	3296	28.3	27.2-29.3
Somewhat approve	18	1.1	0.6-1.8	119	1.5	1.2-1.9
Strongly or very strongly approve	12	1.0	0.5-2.1	61	1.0	0.7-1.4
I don't know enough to rate it or I have never heard about it	1382	61.5	58.7-64.2	7108	69.3	68.1-70.3
Adjusted prevalence difference (95% CI)	2.37 (-2.47, 7.22)			Referent		

Note: Adjusted models include age, gender, race and ethnicity, income, education, region, political party, and potential draftee. Adjusted differences are for the strongly or very strongly approve comparison. Significance is indicated by q-values, which represent the probability that the given difference would be a false discovery, or the expected proportion of "false positives" that would be seen among the collection of all differences whose q-values were at or below the given q-value.

Statement	Unweighted N	Weighted %	CI	Unweighted N	Weighted %	CI
Do you believe that things in this country today are						
Generally headed in the wrong direction	953	85.3	82.0-88.1	969	83.4	80.4-86.1
Generally headed in the right direction	147	14.7	11.9-18.0	173	16.6	13.9-19.6
Adjusted prevalence difference (95% CI)		1.31 (-2.80, 5.42)			Referent	
When thinking about democracy in the United States these days do you believe						
There is a serious threat to our democracy	846	73.5	69.4-77.3	879	75.3	71.8-78.4
There may be a threat to our democracy, but it is not serious	208	22.5	18.8-26.6	211	19.9	17.1-23.0
There is no threat to our democracy	49	4.0	3.0-5.5	56	4.9	3.3-7.1
Adjusted prevalence difference (95% CI)		-0.44 (-5.28, 4.41)			Referent	
How important do you think it is for the United States to remain a democracy?						
Not important	10	2.0	0.7-5.8	12	1.5	0.8-2.7
Somewhat important	20	2.5	1.5-4.2	32	3.6	2.4-5.4
Very or extremely important	1075	95.5	92.2-97.4	1102	94.9	92.9-96.4
Adjusted prevalence difference (95% CI)		-0.57 (-2.22, 1.07)[a]			Referent	
Democracy is the best form of government.						
Do not agree	34	3.6	2.3-5.4	40	4.4	3.0-6.4
Somewhat agree	129	14.9	11.8-18.7	156	17.2	14.4-20.5
Strongly or very strongly agree	940	81.6	77.7-84.9	945	78.4	74.9-81.5
Adjusted prevalence difference (95% CI)		-0.29 (-2.37, 1.79)			Referent	
These days, American democracy only serves the interests of the wealthy and powerful.						
Do not agree	418	34.1	30.5-37.8	455	34.1	30.8-37.6
Somewhat agree	363	34.5	30.5-38.8	376	35.0	31.4-38.6
Strongly or very strongly agree	322	31.4	27.7-35.3	314	30.9	27.3-34.8
Adjusted prevalence difference (95% CI)		-0.82 (-6.08, 4.43)			Referent	
Having a strong leader for America is more important than having a democracy.						
Do not agree	752	63.2	58.7-67.4	766	63.3	59.5-67.0
Somewhat agree	170	19.9	16.1-24.4	192	17.7	14.9-20.9
Strongly or very strongly agree	176	16.9	13.9-20.4	183	19.0	16.0-22.4
Adjusted prevalence difference (95% CI)		-0.70 (-5.07, 3.66)			Referent	
The 2020 election was stolen from Donald Trump, and Joe Biden is an illegitimate president.						
Do not agree	652	58.1	54.0-62.1	712	64.4	60.7-67.9
Somewhat agree	207	19.3	16.3-22.6	174	14.0	11.6-16.7
Strongly or very strongly agree	235	22.7	19.3-26.4	247	21.6	18.7-24.9
Adjusted prevalence difference (95% CI)		-0.06 (-3.92, 3.80)			Referent	
Armed citizens should patrol polling places at election time.						
Do not agree	973	85.7	81.9-88.9	1005	88.0	85.5-90.2
Somewhat agree	74	8.5	5.9-12.2	88	7.7	6.0-9.8
Strongly or very strongly agree	48	5.7	4.0-8.1	46	4.3	3.0-6.1
Adjusted prevalence difference (95% CI)		1.85 (-0.73, 4.43)			Referent	

Note: Adjusted models include age, gender, race and ethnicity, income, education, region, political party, and potential draftee. Adjusted differences are for the first response option comparison for the initial four statements and for the strongly or very strongly agree comparison for the remaining statements. Significance is indicated by q-values, which represent the probability that the given difference would be a false discovery, or the expected proportion of "false positives" that would be seen among the collection of all differences whose q-values were at or below the given q-value.

[a]Political party variable was excluded as it is a sparse indicator.

Table S14. Beliefs Concerning the Potential Need for Violence in the USA by Combat Experience.

Statement	Combat experience			Military background without combat experience		
	Unweighted N	Weighted %	CI	Unweighted N	Weighted %	CI
If elected leaders will not protect American democracy, the people must do it themselves, even if it requires taking violent actions.						
Do not agree	488	40.9	37.1-44.8	528	47.8	44.0-51.6
Somewhat agree	328	33.0	29.0-37.3	364	31.2	27.9-34.7
Strongly or very strongly agree	285	26.1	22.7-29.8	245	21.0	18.1-24.2
Adjusted prevalence difference (95% CI)	3.79 (-0.62, 8.20)			Referent		
Our American way of life is disappearing so fast that we may have to use force to save it.						
Do not agree	619	52.5	48.3-56.6	659	57.5	53.7-61.2
Somewhat agree	298	29.8	25.9-33.9	313	26.8	23.6-30.3
Strongly or very strongly agree	179	17.8	14.7-21.2	167	15.7	13.2-18.7
Adjusted prevalence difference (95% CI)	2.54 (-1.33, 6.41)			Referent		
Because things have gotten so far off track, true American patriots may have to resort to violence in order to save our country.						
Do not agree	784	67.8	63.4-71.8	825	71.8	68.1-75.2
Somewhat agree	222	22.4	18.8-26.5	231	21.0	17.9-24.5
Strongly or very strongly agree	91	9.8	7.4-12.9	78	7.2	5.5-9.5
Adjusted prevalence difference (95% CI)	2.63 (-0.55, 5.81)			Referent		
In the next few years there will be civil war in the United States.						
Do not agree	584	49.4	45.4-53.5	616	50.4	46.6-54.2
Somewhat agree	382	37.5	33.4-41.7	391	36.1	32.5-39.8
Strongly or very strongly agree	131	13.1	10.5-16.3	129	13.6	10.9-16.7
Adjusted prevalence difference (95% CI)	2.96 (-1.07, 7.00)			Referent		

Note: Adjusted models include age, gender, race and ethnicity, income, education, region, political party, and potential draftee. Adjusted differences are for the strongly or very strongly agree comparison. Significance is indicated by q-values, which represent the probability that the given difference would be a false discovery, or the expected proportion of "false positives" that would be seen among the collection of all differences whose q-

Table S15. Beliefs Concerning Race and Ethnicity and American Society by Combat Experience.

Statement	Combat experience			Military background without combat experience		
	Unweighted N	Weighted %	CI	Unweighted N	Weighted %	CI
White people benefit from advantages in society that Black people do not have.						
Do not agree	471	42.7	38.7-46.8	504	41.1	37.5-44.8
Somewhat agree	334	27.6	24.4-31.0	319	26.5	23.3-29.8
Strongly or very strongly agree	297	29.7	25.8-34.0	317	32.5	28.8-36.3
Adjusted prevalence difference (95% CI)		-2.93 (-7.49, 1.62)			Referent	
Straight white men hold far too much power in America.						
Do not agree	628	53.8	49.6-57.9	661	53.5	49.6-57.3
Somewhat agree	251	22.7	19.3-26.5	253	22.0	19.0-25.4
Strongly or very strongly agree	218	23.5	19.8-27.6	219	24.5	21.0-28.4
Adjusted prevalence difference (95% CI)		0.34 (-4.28, 4.95)			Referent	
Discrimination against whites is as big a problem as discrimination against Blacks and other minorities.						
Do not agree	435	38.9	35.0-42.9	467	43.1	39.3-46.9
Somewhat agree	292	26.4	22.7-30.4	296	24.6	21.5-28.0
Strongly or very strongly agree	376	34.7	31.0-38.6	376	32.3	29.0-35.8
Adjusted prevalence difference (95% CI)		2.02 (-2.28, 6.32)			Referent	
Having more Black Americans, Latinos, and Asian Americans is good for the country.						
Do not agree	267	23.9	20.4-27.8	302	23.5	20.6-26.7
Somewhat agree	421	35.0	31.4-38.7	442	38.3	34.7-42.0
Strongly or very strongly agree	399	41.1	37.0-45.4	386	38.2	34.4-42.1
Adjusted prevalence difference (95% CI)		1.21 (-3.67, 6.08)			Referent	
In America, native-born white people are being replaced by immigrants.						
Do not agree	570	51.6	47.5-55.6	560	53.1	49.4-56.9
Somewhat agree	334	28.9	25.5-32.5	381	31.4	28.0-35.0
Strongly or very strongly agree	196	19.6	16.4-23.2	197	15.5	13.2-18.1
Adjusted prevalence difference (95% CI)		3.87 (-0.00, 7.75)			Referent	

Note: Adjusted models include age, gender, race and ethnicity, income, education, region, political party, and potential draftee. Adjusted differences are for the strongly or very strongly agree comparison. Significance is indicated by q-values, which represent the probability that the given difference would be a false discovery, or the expected proportion of “false positives” that would be seen among the collection of all differences [illegible]

Table S16. Beliefs Concerning Qanon and the Biblical "End Times" by Combat Experience.

Statement	Combat experience			Military background without combat experience		
	Unweighted N	Weighted %	CI	Unweighted N	Weighted %	CI
The government, media, and financial worlds in the U.S. are controlled by a group of Satan-worshipping pedophiles who run a global child sex trafficking operation.						
Do not agree	911	79.0	74.9-82.6	953	80.5	77.0-83.5
Somewhat agree	109	12.3	9.5-15.8	113	11.5	9.0-14.6
Strongly or very strongly agree	69	8.7	6.3-11.8	68	8.0	6.1-10.4
Adjusted prevalence difference (95% CI)	-0.29 (-3.33, 2.76)			Referent		
There is a storm coming soon that will sweep away the elites in power and restore the rightful leaders.						
Do not agree	785	69.0	64.8-72.9	811	70.3	66.6-73.7
Somewhat agree	215	22.1	18.5-26.3	223	19.3	16.5-22.5
Strongly or very strongly agree	90	8.9	7.0-11.3	101	10.4	8.1-13.2
Adjusted prevalence difference (95% CI)	0.26 (-2.96, 3.47)			Referent		
The chaos in America today is evidence that we are living in what the Bible calls “the end times.”						
Do not agree	675	57.1	52.8-61.2	672	57.2	53.3-60.9
Somewhat agree	277	28.0	24.0-32.2	279	24.5	21.3-28.0
Strongly or very strongly agree	145	15.0	12.3-18.2	185	18.4	15.5-21.6
Adjusted prevalence difference (95% CI)	-1.84 (-6.54, 2.87)			Referent		

Note: Adjusted models include age, gender, race and ethnicity, income, education, region, political party, and potential draftee. Adjusted differences are for the strongly or very strongly agree comparison. Significance is indicated by q-values, which represent the probability that the given difference would be a false discovery, or the expected proportion of “false positives” that would be seen among the collection of all differences whose q-[illegible]

Table S17. Justification for Violence in Non-Political Situations by Combat Experience.

What do you think about the use of force or violence in the following situations?	Combat experience			Military background without combat experience		
	Unweighted N	Weighted %	CI	Unweighted N	Weighted %	CI
In self defense						
Never justified	7	0.58	0.3-1.2	9	0.97	0.4-2.1
Sometimes justified	156	14.97	12.0-18.6	142	13.19	10.7-16.1
Usually or always justified	941	84.45	80.8-87.5	994	85.84	82.9-88.4
Adjusted prevalence difference (95% CI)		-1.82 (-5.83, 2.20)			Referent	
To prevent someone from injuring or killing another person						
Never justified	6	0.65	0.3-1.5	9	1.01	0.5-2.2
Sometimes justified	112	10.86	8.2-14.3	110	11.51	9.0-14.6
Usually or always justified	979	88.49	85.1-91.2	1020	87.48	84.4-90.1
Adjusted prevalence difference (95% CI)		0.14 (-3.57, 3.84)			Referent	
To prevent someone from injuring or killing themselves						
Never justified	56	6.09	4.0-9.1	60	6.64	4.9-9.0
Sometimes justified	363	33.59	29.9-37.5	333	29.89	26.4-33.6
Usually or always justified	680	60.33	56.2-64.3	750	63.47	59.7-67.1
Adjusted prevalence difference (95% CI)		-4.39 (-9.67, 0.89)			Referent	
To prevent harm or damage to property						
Never justified	108	11.08	8.5-14.3	133	12.15	9.9-14.8
Sometimes justified	528	47.99	43.9-52.1	555	48.2	44.4-52.0
Usually or always justified	467	40.93	37.0-45.0	454	39.65	36.0-43.4
Adjusted prevalence difference (95% CI)		1.52 (-3.73, 6.76)			Referent	
To win an argument						
Never justified	997	88.55	84.5-91.6	1040	89.62	86.8-91.9
Sometimes justified	81	7.75	5.5-10.9	80	7.6	5.7-10.1
Usually or always justified	20	3.7	1.8-7.3	23	2.79	1.7-4.5
Adjusted prevalence difference (95% CI)		0.99 (-1.19, 3.18)			Referent	
In response to an insult						
Never justified	975	85.31	81.3-88.6	1024	87.56	84.4-90.2
Sometimes justified	98	9.89	7.4-13.1	93	9.62	7.3-12.6
Usually or always justified	27	4.8	2.7-8.3	26	2.82	1.8-4.5
Adjusted prevalence difference (95% CI)		1.89 (-0.89, 4.67)			Referent	
To get respect						
Never justified	1023	90.93	87.5-93.5	1055	90.02	87.1-92.4
Sometimes justified	51	4.93	3.4-7.1	63	7.22	5.2-10.0
Usually or always justified	27	4.13	2.2-7.5	25	2.75	1.7-4.4
Adjusted prevalence difference (95% CI)		0.96 (-0.95, 2.86)			Referent	

Note: Adjusted models include age, gender, race and ethnicity, income, education, region, political party, and potential draftee. Adjusted differences are for the usually or always justified comparison. [illegible]

Table S18. Justification for Political Violence, in General and for 9 Specific Objectives by Combat Experience.

What do you think about the use of force or violence in the following situations?	Combat experience			Military background without combat experience		
	Unweighted N	Weighted %	CI	Unweighted N	Weighted %	CI
In general what do you think about using force or violence to advance an important political objective that you support						
Never justified	939	80.76	76.5-84.4	984	84.22	81.0-87.0
Sometimes justified	150	17.39	13.8-21.7	154	15.09	12.3-18.3
Usually or always justified	15	1.85	1.0-3.3	8	0.69	0.3-1.5
Adjusted prevalence difference (95% CI)		0.71 (-0.41, 1.82)			Referent	
Thinks violence is usually or always justified to advance at least 1 of 17 objectives	463	40.11	36.1-44.2	466	38.43	34.9-42.1
Adjusted prevalence difference (95% CI)		0.69 (-4.31, 5.70)			Referent	
To return Donald Trump to the presidency this year						
Never justified	1010	90.04	86.3-92.8	1048	91.31	88.6-93.4
Sometimes justified	38	3.57	2.4-5.2	41	4.11	2.7-6.3
Usually or always justified	46	6.38	3.9-10.2	48	4.59	3.2-6.5
Adjusted prevalence difference (95% CI)		0.02 (-2.69, 2.73)			Referent	
To stop an election from being stolen						
Never justified	787	70.97	66.9-74.8	816	73.22	69.8-76.4
Sometimes justified	191	17.05	14.2-20.4	209	17.56	14.8-20.7
Usually or always justified	114	11.98	9.2-15.5	108	9.22	7.4-11.5
Adjusted prevalence difference (95% CI)		1.51 (-1.76, 4.77)			Referent	
To stop people who do not share my beliefs from voting						
Never justified	1062	94.81	91.6-96.8	1111	96.35	93.9-97.8
Sometimes justified	23	2.49	1.5-4.1	18	2.85	1.5-5.4
Usually or always justified	14	2.7	1.2-6.2	9	0.8	0.4-1.7
Adjusted prevalence difference (95% CI)		1.00 (-0.42, 2.42)			Referent	
To prevent discrimination based on race or ethnicity						
Never justified	710	63.52	59.4-67.4	756	64.55	60.8-68.2
Sometimes justified	301	27.51	24.2-31.1	304	26.86	23.6-30.4
Usually or always justified	83	8.96	6.4-12.4	79	8.59	6.5-11.3
Adjusted prevalence difference (95% CI)		0.22 (-2.86, 3.31)			Referent	
To preserve an American way of life based on Western European traditions						
Never justified	740	68.11	64.1-71.9	755	68.83	65.3-72.1
Sometimes justified	274	26.26	22.6-30.2	281	23.06	20.1-26.3
Usually or always justified	74	5.63	4.3-7.3	98	8.12	6.4-10.3
Adjusted prevalence difference (95% CI)		-2.77 (-5.09, -0.46)			Referent	
To preserve the American way of life I believe in						
Never justified	444	41.8	37.8-45.9	471	44.23	40.5-48.1
Sometimes justified	454	42.2	38.1-46.4	465	38.3	34.7-42.0
Usually or always justified	201	15.99	13.6-18.7	209	17.47	14.9-20.3
Adjusted prevalence difference (95% CI)		-2.09 (-5.87, 1.69)			Referent	
To oppose Americans who do not share my beliefs						
Never justified	1012	91.28	88.2-93.6	1063	91.85	89.2-93.9
Sometimes justified	65	6.15	4.1-9.1	60	6.25	4.4-8.8
Usually or always justified	23	2.57	1.6-4.1	22	1.9	1.1-3.2
Adjusted prevalence difference (95% CI)		0.51 (-1.03, 2.06)			Referent	
To oppose the government when it does not share my beliefs						
Never justified	911	80.19	76.1-83.8	939	82.35	79.2-85.1
Sometimes justified	159	17.17	13.7-21.3	171	14.98	12.4-18.0
Usually or always justified	26	2.63	1.7-4.1	27	2.67	1.7-4.2
Adjusted prevalence difference (95% CI)		-0.28 (-2.10, 1.55)			Referent	
To oppose the government when it tries to take private land for public purposes						
Never justified	656	58.8	54.7-62.8	677	56.98	53.1-60.7
Sometimes justified	342	30.92	27.4-34.7	366	33.12	29.5-36.9
Usually or always justified	100	10.28	7.7-13.7	92	9.9	7.8-12.5
Adjusted prevalence difference (95% CI)		0.24 (-2.89, 3.36)			Referent	

Note: Adjusted models include age, gender, race and ethnicity, income, education, region, political party, and potential draftee. Adjusted differences are for the usually or always justified comparison. Significance is indicated by q-values, which represent the probability that the given difference would be a false discovery, or the expected proportion of "false positives" that would be seen among the collection

Table S19. Justification for Political Violence for 8 Additional Specific Objectives by Combat Experience (these objectives were paired, with respondents randomized 1:1 to see 1 item in each pair).

What do you think about the use of force or violence in the following situations?	Combat experience			Military background without combat experience		
	Unweighted N	Weighted %	CI	Unweighted N	Weighted %	CI
To stop voter fraud						
Never justified	400	69.31	63.3-74.7	398	70.31	65.2-75.0
Sometimes justified	102	16.69	12.9-21.4	97	17.45	13.7-22.0
Usually or always justified	70	14	9.7-19.7	66	12.24	9.2-16.1
Adjusted prevalence difference (95% CI)	-0.20 (-4.80, 4.40)			Referent		
To stop voter intimidation						
Never justified	264	53.14	47.4-58.8	321	60.16	55.0-65.1
Sometimes justified	184	33.09	28.2-38.4	191	29.29	24.8-34.2
Usually or always justified	80	13.77	10.3-18.1	72	10.55	8.0-13.8
Adjusted prevalence difference (95% CI)	3.21 (-1.96, 8.38)			Referent		
To reinforce the police						
Never justified	143	27.05	22.3-32.4	153	31.02	26.2-36.3
Sometimes justified	266	48.13	42.5-53.8	265	40.47	35.6-45.6
Usually or always justified	161	24.82	20.7-29.5	187	28.51	24.4-33.1
Adjusted prevalence difference (95% CI)	-4.70 (-10.63, 1.24)			Referent		
To stop police violence						
Never justified	266	47.62	41.8-53.6	285	48.86	43.3-54.4
Sometimes justified	204	39.14	33.3-45.3	205	41.38	35.9-47.1
Usually or always justified	58	13.23	9.0-19.0	49	9.76	7.1-13.4
Adjusted prevalence difference (95% CI)	2.83 (-1.57, 7.24)			Referent		
To stop illegal immigration						
Never justified	240	49.27	42.6-56.0	283	52.29	47.1-57.4
Sometimes justified	177	35.77	29.8-42.3	192	32.12	27.5-37.2
Usually or always justified	85	14.96	10.7-20.6	96	15.59	12.4-19.4
Adjusted prevalence difference (95% CI)	-1.82 (-6.92, 3.29)			Referent		
To keep our borders open						
Never justified	430	73.03	68.6-77.0	408	72.87	67.8-77.4
Sometimes justified	110	17.83	14.5-21.8	124	21.19	17.0-26.1
Usually or always justified	57	9.15	6.8-12.2	41	5.94	4.2-8.4
Adjusted prevalence difference (95% CI)	3.42 (0.19, 6.66)			Referent		
To stop a protest or demonstration						
Never justified	322	55.17	49.5-60.7	303	54.71	49.6-59.7
Sometimes justified	228	40.01	34.5-45.8	247	40.4	35.5-45.5
Usually or always justified	32	4.82	3.2-7.1	32	4.89	3.3-7.2
Adjusted prevalence difference (95% CI)	0.09 (-2.65, 2.83)			Referent		
To support a protest or demonstration						
Never justified	433	80.74	74.9-85.5	481	80.99	75.7-85.4
Sometimes justified	68	15.08	10.7-20.8	67	15.47	11.4-20.7
Usually or always justified	17	4.18	2.4-7.1	16	3.54	2.0-6.3
Adjusted prevalence difference (95% CI)	0.21 (-2.24, 2.67)			Referent		

Note: Adjusted models include age, gender, race and ethnicity, income, education, region, political party, and potential draftee. Adjusted differences are for the usually or always justified [illegible]

Table S20. Personal Willingness to Engage in Political Violence Based on the Type of Violence by Combat Experience.

In a situation where you think force or violence is justified to advance an important political objective…How willing would you personally be to use force or violence in each of these ways?	Combat experience			Military background without combat experience		
	Unweighted N	Weighted %	CI	Unweighted N	Weighted %	CI
To damage property						
Not asked the question	149	13.9	11.4-16.8	173	17.7	14.7-21.0
Not willing	859	75.4	71.5-79.0	866	71.8	68.0-75.3
Somewhat willing	71	8.2	5.6-11.8	81	8.4	6.2-11.3
Very or completely willing	21	2.5	1.5-4.0	25	2.2	1.3-3.7
Adjusted prevalence difference (95% CI)		0.61 (-1.14, 2.36)			Referent	
To threaten or intimidate a person						
Not asked the question	149	13.9	11.4-16.8	173	17.7	14.8-21.0
Not willing	845	73.4	69.3-77.2	881	74.0	70.3-77.4
Somewhat willing	85	10.1	7.1-14.1	74	7.0	5.1-9.5
Very or completely willing	21	2.6	1.6-4.2	14	1.3	0.7-2.4
Adjusted prevalence difference (95% CI)		1.04 (-0.59, 2.68)			Referent	
To injure a person						
Not asked the question	149	13.9	11.4-16.8	173	17.7	14.8-21.1
Not willing	853	75.2	71.3-78.7	878	73.2	69.5-76.7
Somewhat willing	74	8.2	5.8-11.4	72	7.7	5.6-10.3
Very or completely willing	23	2.7	1.7-4.4	18	1.4	0.8-2.4
Adjusted prevalence difference (95% CI)		1.21 (-0.46, 2.87)			Referent	
To kill a person						
Not asked the question	149	13.9	11.5-16.8	173	17.7	14.8-21.0
Not willing	871	78.0	74.6-81.1	910	76.5	72.8-79.8
Somewhat willing	57	5.8	4.2-8.0	41	4.5	2.9-6.9
Very or completely willing	20	2.3	1.4-3.7	19	1.3	0.8-2.3
Adjusted prevalence difference (95% CI)		1.06 (-0.52, 2.65)			Referent	

Note: Adjusted models include age, gender, race and ethnicity, income, education, region, political party, and potential draftee. Adjusted differences are for the very or completely willing comparison. Significance is indicated by q-values, which represent the probability that the given difference would be a false discovery, or the expected proportion of "false positives" that would be seen among the collection of all differences whose q-values were at or below the given q-value.

Table S21. Personal Willingness to Engage in Political Violence Based on the Target of Violence by Combat Experience.

In a situation where you think force or violence is justified to advance an important political objective…How willing would you personally be to use force or violence against a person because they are…	Combat experience			Military background without combat experience		
	Unweighted N	Weighted %	CI	Unweighted N	Weighted %	CI
An elected federal or state government official						
Not asked the question	149	13.9	11.5-16.8	173	17.8	14.9-21.2
Not willing	858	75.4	71.2-79.1	897	76.1	72.5-79.5
Somewhat willing	73	7.9	5.5-11.1	57	5.4	3.7-7.8
Very or completely willing	18	2.9	1.3-6.3	10	0.7	0.3-1.4
Adjusted prevalence difference (95% CI)	1.28 (0.01, 2.56)				Referent	
An elected local government official						
Not asked the question	149	13.9	11.5-16.8	173	17.8	14.8-21.1
Not willing	880	77.1	72.9-80.7	902	76.6	73.0-79.9
Somewhat willing	56	7.7	4.9-11.8	53	4.5	3.0-6.6
Very or completely willing	13	1.3	0.7-2.5	11	1.2	0.5-2.5
Adjusted prevalence difference (95% CI)	0.57 (-0.73, 1.88)				Referent	
An election worker such as a poll worker or vote counter						
Not asked the question	149	13.8	11.4-16.7	173	17.8	14.9-21.2
Not willing	915	80.7	76.9-83.9	934	79.2	75.6-82.4
Somewhat willing	27	3.8	2.0-7.3	26	2.6	1.4-4.7
Very or completely willing	12	1.7	0.9-3.1	6	0.5	0.2-1.2
Adjusted prevalence difference (95% CI)	1.70 (0.21, 3.18)				Referent	
A public health official						
Not asked the question	149	13.9	11.4-16.7	173	17.9	14.9-21.2
Not willing	900	79.3	75.6-82.7	922	78.2	74.6-81.4
Somewhat willing	39	3.8	2.5-5.6	35	3.4	2.1-5.6
Very or completely willing	14	3.0	1.3-6.7	7	0.6	0.2-1.4
Adjusted prevalence difference (95% CI)	2.84 (0.61, 5.06)				Referent	
A member of the military or National Guard						
Not asked the question	149	13.8	11.4-16.7	173	17.8	14.9-21.2
Not willing	887	76.5	72.4-80.2	913	76.5	72.8-79.8
Somewhat willing	45	4.9	3.4-6.9	38	4.3	2.8-6.6
Very or completely willing	21	4.7	2.4-9.1	13	1.4	0.7-2.9
Adjusted prevalence difference (95% CI)	2.81 (0.16, 5.46)				Referent	
A police officer						
Not asked the question	149	13.9	11.4-16.8	173	17.8	14.9-21.2
Not willing	885	78.1	74.4-81.5	911	76.8	73.2-80.2
Somewhat willing	50	5.7	3.6-8.8	41	4.0	2.5-6.2
Very or completely willing	16	2.3	1.3-4.0	12	1.3	0.6-2.7
Adjusted prevalence difference (95% CI)	1.85 (-0.06, 3.77)				Referent	
A person who does not share your race or ethnicity						
Not asked the question	149	13.8	11.4-16.7	173	17.8	14.8-21.1
Not willing	918	81.0	77.3-84.2	935	78.6	74.9-81.8
Somewhat willing	26	3.1	1.9-4.9	22	2.7	1.4-4.9
Very or completely willing	10	2.1	0.7-5.9	9	1.0	0.4-2.4
Adjusted prevalence difference (95% CI)	0.57 (-0.65, 1.78)[a]				Referent	
A person who does not share your religion						
Not asked the question	149	13.9	11.4-16.8	173	17.8	14.8-21.1
Not willing	923	82.1	78.5-85.2	941	78.7	75.1-82.0
Somewhat willing	15	1.6	0.9-2.9	20	3.0	1.6-5.4
Very or completely willing	11	2.4	0.9-6.1	5	0.5	0.2-1.3
Adjusted prevalence difference (95% CI)	1.70 (0.05, 3.35)				Referent	
A person who does not share your political beliefs						
Not asked the question	149	13.9	11.4-16.7	173	17.8	14.9-21.2
Not willing	902	80.5	77.2-83.4	924	77.8	74.1-81.0
Somewhat willing	40	4.4	3.0-6.4	35	3.6	2.2-5.8
Very or completely willing	10	1.2	0.6-2.5	6	0.9	0.3-2.4
Adjusted prevalence difference (95% CI)	0.62 (-0.61, 1.86)				Referent	

Note: Adjusted models include age, gender, race and ethnicity, income, education, region, political party, and potential draftee. Adjusted differences are for the very or completely willing comparison. Significance is indicated by q-values, which represent the probability that the given difference would be a false discovery, or the expected proportion of "false positives" that would be seen among the collection of all differences whose q-values were at or below the given q-value.

Table S22. Personal Willingness to Use Political Violence as an Individual or Part of a Group by Combat Experience.

You agreed that the use of force or violence could be justified to advance one/some of the political objectives we just discussed. In that/those situation/situations, how willing would you personally be to…	Combat experience			Military background without combat experience		
	Unweighted N	Weighted %	CI	Unweighted N	Weighted %	CI
Use force or violence on your own as an individual						
Not asked the question	149	13.9	11.4-16.7	173	17.7	14.8-21.1
Not willing	640	55.8	51.7-59.9	662	55.3	51.5-59.1
Somewhat willing	228	21.3	18.0-25.0	235	19.4	16.6-22.6
Very or completely willing	84	9.1	6.7-12.3	72	7.5	5.6-10.0
Adjusted prevalence difference (95% CI)	2.40 (-0.70, 5.50)			Referent		
Use force or violence as part of a group of people who share your beliefs						
Not asked the question	149	13.9	11.4-16.7	173	17.8	14.9-21.2
Not willing	750	64.0	59.8-68.0	771	65.3	61.5-68.9
Somewhat willing	163	18.2	14.6-22.3	170	14.9	12.3-18.0
Very or completely willing	39	4.0	2.8-5.6	26	2.0	1.3-3.1
Adjusted prevalence difference (95% CI)	1.87 (0.19, 3.54)			Referent		
Organize a group of people who share your beliefs to use force or violence						
Not asked the question	149	13.8	11.4-16.7	173	17.8	14.8-21.1
Not willing	804	69.4	65.2-73.3	867	73.2	69.5-76.6
Somewhat willing	114	11.6	8.8-15.0	82	7.2	5.4-9.6
Very or completely willing	36	5.2	3.2-8.3	18	1.8	1.0-3.3
Adjusted prevalence difference (95% CI)	2.76 (0.77, 4.76)			Referent		

Note: Adjusted models include age, gender, race and ethnicity, income, education, region, political party, and potential draftee. Adjusted differences are for the very or completely [illegible]

Table S23. Future Likelihood of Firearm Possession and Use in a Situation Where Political Violence is Perceived as Justified by Combat Experience.

Thinking now about the future and all the changes it might bring, how likely is it that you will use a gun in any of the following ways in the next few years—in a situation where you think force or violence is justified to advance an important political objective?	Combat experience			Military background without combat experience		
	Unweighted N	Weighted %	CI	Unweighted N	Weighted %	CI
I will be armed with a gun.						
Not likely	856	73.87	69.4-77.9	904	78.27	74.8-81.4
Somewhat likely	115	11.82	9.2-15.1	109	9.11	7.2-11.4
Very or extremely likely	118	14.31	11.0-18.5	127	12.62	10.0-15.7
Adjusted prevalence difference (95% CI)	-0.74 (-4.50, 3.01)				Referent	
I will carry a gun openly, so that people know I am armed.						
Not likely	966	84.93	80.6-88.4	997	86.32	83.1-89.0
Somewhat likely	73	7.05	5.1-9.6	83	6.56	5.1-8.4
Very or extremely likely	52	8.02	5.1-12.4	55	7.12	5.0-10.1
Adjusted prevalence difference (95% CI)	0.28 (-2.87, 3.44)				Referent	
I will threaten someone with a gun.						
Not likely	1066	94.6	89.9-97.2	1117	98.23	96.8-99.0
Somewhat likely	17	3.39	1.5-7.5	10	0.64	0.3-1.3
Very or extremely likely	9	2.01	0.7-5.9	9	1.13	0.5-2.6
Adjusted prevalence difference (95% CI)	0.08 (-1.52, 1.68)				Referent	
I will shoot someone with a gun.						
Not likely	1045	95.08	92.7-96.7	1097	96.12	94.1-97.5
Somewhat likely	31	3.36	2.0-5.7	31	3.03	1.8-5.0
Very or extremely likely	16	1.55	0.9-2.8	10	0.84	0.4-1.8
Adjusted prevalence difference (95% CI)	0.72 (-0.27, 1.71)				Referent	

Table S24. Approval of Extremist Organizations and Movements by Combat Experience.

How much do you approve or disapprove of these named groups and organizations?	Combat experience			Military background without combat experience		
	Unweighted N	Weighted %	CI	Unweighted N	Weighted %	CI
Proud Boys						
Do not approve	685	60.1	55.93-64.07	680	60.0	56.2-63.6
Somewhat approve	54	5.0	3.67-6.64	64	5.2	3.9-7.1
Strongly or very strongly approve	22	2.9	1.78-4.67	20	2.0	1.1-3.4
I don't know enough to rate it or I have never heard about it	339	32.1	28.24-36.21	380	32.8	29.4-36.5
Adjusted prevalence difference (95% CI)	1.20 (-1.29, 3.69)				Referent	
Oath Keepers						
Do not approve	555	48.4	44.33-52.49	518	44.3	40.5-48.0
Somewhat approve	64	5.3	4.05-7.03	57	4.5	3.3-6.1
Strongly or very strongly approve	44	4.4	3.08-6.17	46	4.1	2.8-5.8
I don't know enough to rate it or I have never heard about it	438	41.9	37.82-46.06	520	47.2	43.4-51.0
Adjusted prevalence difference (95% CI)	-0.46 (-3.95, 3.04)				Referent	
Three Percenters						
Do not approve	393	34.4	30.78-38.30	382	34.0	30.5-37.7
Somewhat approve	27	2.8	1.85-4.32	29	2.4	1.5-3.6
Strongly or very strongly approve	17	1.6	0.93-2.90	14	1.8	0.9-3.3
I don't know enough to rate it or I have never heard about it	664	61.1	57.13-64.89	715	61.9	58.1-65.5
Adjusted prevalence difference (95% CI)	0.32 (-4.04, 4.67)				Referent	
Militia movement						
Do not approve	608	52.3	48.17-56.41	633	54.0	50.2-57.8
Somewhat approve	72	8.6	6.07-11.95	71	5.4	4.0-7.2
Strongly or very strongly approve	30	3.3	2.00-5.30	25	2.7	1.7-4.3
I don't know enough to rate it or I have never heard about it	388	35.9	32.01-39.92	413	37.9	34.2-41.8
Adjusted prevalence difference (95% CI)	2.96 (-0.79, 6.71)				Referent	
White supremacy movement						
Do not approve	919	82.7	79.17-85.80	951	83.8	81.0-86.2
Somewhat approve	13	1.8	0.92-3.40	24	2.4	1.5-3.8
Strongly or very strongly approve	11	1.1	0.57-2.19	5	0.7	0.2-2.3
I don't know enough to rate it or I have never heard about it	157	14.4	11.52-17.78	161	13.1	11.0-15.6
Adjusted prevalence difference (95% CI)	0.81 (-0.83, 2.45)[a]				Referent	
Christian nationalist movement						
Do not approve	450	38.7	34.91-42.71	430	38.8	35.2-42.6
Somewhat approve	90	9.0	6.71-11.85	101	8.2	6.5-10.3
Strongly or very strongly approve	42	5.1	3.29-7.75	54	5.1	3.6-7.2
I don't know enough to rate it or I have never heard about it	516	47.2	43.16-51.35	556	47.9	44.1-51.7
Adjusted prevalence difference (95% CI)	-2.23 (-7.56, 3.10)				Referent	
Boogaloo movement						
Do not approve	417	36.5	32.81-40.43	407	36.4	32.9-40.1
Somewhat approve	8	1.2	0.54-2.74	10	1.0	0.5-2.0
Strongly or very strongly approve	7	1.0	0.43-2.19	5	1.0	0.4-2.7
I don't know enough to rate it or I have never heard about it	666	61.3	57.32-65.09	716	61.6	57.8-65.2
Adjusted prevalence difference (95% CI)	0.06 (-4.55, 4.68)[b]				Referent	

Note: Adjusted models include age, gender, race and ethnicity, income, education, region, political party, and potential draftee. Adjusted differences are for the strongly or very strongly approve comparison. Significance is indicated by q-values, which represent the probability that the given difference would be a false discovery, or the expected proportion of "false positives" that would be seen among the collection of all differences whose q-values were at or below the given q-value.

$50,000 to $74,999	179	16.8	13.8-20.3	148	16.3	13.3-19.8	118	22.1	17.8-27.2	-0.5 (-20.3, 19.2)
$75,000 to $99,999	191	15.4	13.0-18.3	126	15.3	12.2-19.0	97	16.1	12.4-20.5	-3.0 (-22.9, 16.8)
$100,000 to $149,999	219	20.3	17.1-23.9	150	22.5	18.4-27.2	102	18.1	14.0-22.9	-6.3 (-26.1, 13.6)
$150,000 or more	184	23	19.2-27.1	112	19	15.5-23.0	62	16.1	12.0-21.3	-7.6 (-27.6, 12.4)
Political party										
Strong Republican	161	15.4	12.7-18.6	196	25.7	21.9-30.0	216	37.5	32.3-43.0	Referent
Not Strong Republican	112	10.8	8.3-14.0	77	11.2	8.5-14.7	57	11.8	8.4-16.3	-12.4 (-21.0, -3.7)*
Leans Republican	193	19.2	15.7-23.3	168	22.9	18.9-27.4	149	26.8	22.2-31.9	-9.5 (-16.6, -2.5)*
Undecided/Independent/Other	41	4.5	3.1-6.6	21	2.5	1.5-4.2	9	1.4	0.7-2.9	-25.9 (-35.3, -16.5)***
Leans Democrat	202	20.3	16.9-24.2	105	16.4	12.8-20.7	44	9.1	6.1-13.5	-24.9 (-32.0, -17.8)***
Not Strong Democrat	102	11.2	8.7-14.1	46	9.1	6.2-13.1	22	5.5	2.9-10.5	-22.5 (-31.1, -13.8)***
Strong Democrat	204	18.6	15.8-21.8	79	12.2	8.8-16.7	32	7.9	4.7-12.7	-22.4 (-30.1, -14.8)***
Draft										
Not potential draftee	463	59.8	55.7-63.8	368	67.1	62.6-71.3	277	66.3	61.2-71.0	Referent
Potential draftee	553	40.2	36.2-44.3	324	32.9	28.7-37.4	253	33.7	29.0-38.8	1.7 (-6.5, 9.9)

Note: Adjusted prevalence differences compare those who strongly or very strongly agree to those who do not, controlling for other sociodemographic covariates. Significance is indicated by q-values, which represent the probability that the given difference would be a false discovery, or the expected proportion of "false positives" that would be seen among the collection of all differences whose q-values were at or below the given q-value.

*q<.05, ***q≤.001
